# Metabolomics for personalized medicine: the input of analytical chemistry from biomarker discovery to point-of-care tests

**DOI:** 10.1007/s00216-021-03586-z

**Published:** 2021-08-25

**Authors:** Florence Anne Castelli, Giulio Rosati, Christian Moguet, Celia Fuentes, Jose Marrugo-Ramírez, Thibaud Lefebvre, Hervé Volland, Arben Merkoçi, Stéphanie Simon, François Fenaille, Christophe Junot

**Affiliations:** 1 Université Paris-Saclay, CEA, INRAE, Département Médicaments et Technologies pour la Santé (MTS), Gif-sur-Yvette cedex, 91191 France; 2 MetaboHUB, Gif-sur-Yvette, France; 3 Institut Català de Nanociència i Nanotecnologia (ICN2), Edifici ICN2 Campus UAB, 08193 Bellaterra, Barcelona, Spain; 4 Centre de Recherche sur l’Inflammation/CRI, Université de Paris, Inserm, Paris, France; 5 CRMR Porphyrie, Hôpital Louis Mourier, AP-HP Nord - Université de Paris, Colombes, France

**Keywords:** Metabolomics, Biomarkers, Personalized medicine, Precision medicine, Point-of-care tests, Immunoassays, Biosensors

## Abstract

Metabolomics refers to the large-scale detection, quantification, and analysis of small molecules (metabolites) in biological media. Although metabolomics, alone or combined with other omics data, has already demonstrated its relevance for patient stratification in the frame of research projects and clinical studies, much remains to be done to move this approach to the clinical practice. This is especially true in the perspective of being applied to personalized/precision medicine, which aims at stratifying patients according to their risk of developing diseases, and tailoring medical treatments of patients according to individual characteristics in order to improve their efficacy and limit their toxicity. In this review article, we discuss the main challenges linked to analytical chemistry that need to be addressed to foster the implementation of metabolomics in the clinics and the use of the data produced by this approach in personalized medicine. First of all, there are already well-known issues related to untargeted metabolomics workflows at the levels of data production (lack of standardization), metabolite identification (small proportion of annotated features and identified metabolites), and data processing (from automatic detection of features to multi-omic data integration) that hamper the inter-operability and reusability of metabolomics data. Furthermore, the outputs of metabolomics workflows are complex molecular signatures of few tens of metabolites, often with small abundance variations, and obtained with expensive laboratory equipment. It is thus necessary to simplify these molecular signatures so that they can be produced and used in the field. This last point, which is still poorly addressed by the metabolomics community, may be crucial in a near future with the increased availability of molecular signatures of medical relevance and the increased societal demand for participatory medicine.

**Graphical abstract:**

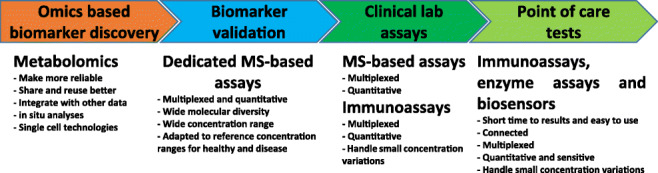

## Introduction

“Omics” analyses are based on the use of large-scale data production techniques (such as nucleic acid sequencing or mass spectrometry). These, coupled with data mining and interpretation tools based on mathematics, statistical analyses, and bioinformatics, make it possible to analyze and understand a system or living organism as a whole, at the different levels of its biological organization (i.e., genomics, transcriptomics, proteomics, and metabolomics for the sets of genes, transcripts, proteins, and metabolites, respectively).

Metabolomics has emerged in the late 1990s, following the development of proteomics [1]. It deals with the detection, identification, and quantification of the small molecular weight compounds present in a given biological medium. The metabolome includes all the compounds of low molecular weight (< 1000 Da or < 1500 Da according to the definitions) that are present in a biological sample, and excludes biological polymers such as proteins or nucleic acids [2]. Such metabolites can be molecules involved in the ubiquitous reactions by which the cells of an organism produce and use energy, as for example amino acids, sugars, nucleotides, or fatty acids. Some other kinds of metabolites can be synthesized by any given biological species for achieving particular biological functions, as for example glucocorticoids or neurotransmitters in mammals, or alkaloids in plants. Xenobiotics (i.e., chemicals which are found in living organisms, but not produced by them) such as drugs and pollutants and their metabolites are also part of the metabolome since they are present in biological media and can be detected by the analytical chemistry tools that are used for metabolomics [3]. This highlights that metabolites are of different origins and come not only from the cellular metabolism, but also from the microbiota, food and drinks, drug intake, and environment. This makes metabolomics an efficient tool to track interactions between a living organism and its environment. However, many metabolites are still uncharacterized due to their large chemical diversity and to insufficient knowledge about metabolism. As a matter of fact, it is estimated that less than 5% of the features detected in biological media using mass spectrometry-based metabolomics methods are annotated [4].

Metabolomics is thus far more complex than the metabolic pathways displayed in metabolic biochemistry textbooks. It is indeed impossible to know exactly how many metabolites compose the metabolomes of living organisms, whereas theoretical proteomes can be more readily inferred from genomic data. Furthermore, as metabolites exhibit a huge chemical diversity, ranging from polar and hydrophilic compounds such as sugar derivatives to apolar and hydrophobic molecules such as lipids, there is no universal method for metabolome analysis. Consequently, the detection of metabolites relies on complementary methods that have to be run in parallel to achieve optimal metabolome coverage [5]. The two main technologies for metabolomics data production are based on nuclear magnetic resonance (NMR) spectroscopy and mass spectrometry (MS). The latter can be used as a standalone technique (i.e., direct introduction mass spectrometry) or coupled with gas chromatography (GC-MS), liquid chromatography (LC-MS), or capillary electrophoresis (CE-MS).

There are two main kinds of metabolomics approaches: targeted and untargeted (or global) ones. Untargeted approaches seek to detect as many compounds as possible in samples. Such compounds can be known metabolites, putatively annotated metabolites, or unknown ones. In this case, metabolite concentrations are given in a semi-quantitative manner (relative quantification). This means that metabolite concentrations are expressed as arbitrary units or ion intensities. In untargeted MS-based metabolomics, metabolite abundances are generally provided in chromatographic peak areas. Those peak areas highly depend on the LC-HRMS conditions and thus remain linked to a dataset and are difficult to be directly compared with those from other metabolomics profiling experiments performed at different time periods or within different laboratories. The most used detection methods for global metabolomics approaches are NMR, GC/MS, and liquid chromatography coupled to high-resolution mass spectrometry (LC-HRMS). These untargeted approaches are used especially in the first steps of biomarker discovery. Conversely, targeted approaches are focused on a limited number of compounds, belonging to a given chemical class or metabolic pathway. They can be more sensitive than untargeted approaches and can provide, if needed, absolute quantification with results expressed in molarity units. They can be used for mechanistic purposes (in the frame of fluxomic experiments, for example) or for biomarker validation following untargeted approaches [6, 7].

Metabolomics workflows, including sample preparation, MS and/or NMR analyses, data pre-processing, statistical analyses, and data visualization, have been developed since the 2000s and have now reached a certain level of maturity. Nowadays, metabolomics is considered as a tool in its own right and is used in systems biology projects in many biological fields, such as environmental research [8], plant science [9, 10], nutrition, animal and human health [11, 12], etc. In particular, this is the case in the field of biomedical research, for which systems biology has given rise to systems medicine, a systems approach to health and diseases paving the way to personalized medicine.

Personalized medicine highlights the importance of the individuals’ characteristics in the response to treatment. This concept has been developed with the improvement of our knowledge, which makes it possible to define pathologies more precisely. According to Leroy Hood, one of the pioneers of this approach, personalized medicine promises to (i) provide deep insights into disease mechanisms, (ii) make blood a diagnostic window for viewing health and disease of an individual, (iii) stratify complex diseases into subtypes, (iv) provide new approaches to drug target discovery, and (v) generate metrics for assessing wellness. Thus, medicine aims at being *p*reventive, *p*redictive, *p*ersonalized, and *p*articipatory, referred to as the concept of “P4 medicine” [13]. This approach is particularly relevant in the field of chronic and non-communicable diseases for which it is often difficult to grasp the transition from the healthy to the disease status. In this context, Sagner et al. have proposed a “P4 health continuum model” with 4 stages of health, namely the A, B, C, and D stages, corresponding respectively to an apparently healthy state, the emergence of chronic disease signs (such as elevated blood pressure or dyslipidemia), the emergence of chronic disease symptoms, and the confirmed chronic disease diagnosis, respectively [14].

Research and development in biomarker discovery are central in modern healthcare for personalized and precision medicine. Omics approaches can be regarded as particularly relevant and useful tools to identify new molecular biomarkers or sets of biomarkers to improve the diagnosis and prognosis of various diseases as well as to evaluate treatment efficacy. In this context, metabolomics represents an attractive strategy for profiling a large panel of low molecular weight molecules in patient samples and for pointing out relevant molecules closely related to (patho)physiological conditions and treatment response phenotypes. However, over the last ten years, the number of biomarkers derived from omics-based approaches, approved by regulatory agencies and used in clinical settings, remains far from expectations [15]. This often makes people perceive metabolomics and other omics as over-promising and/or under-delivering approaches when applied to clinical questions [16]. Despite the publication in the last two decades of more than 2000 scientific papers using MS-based metabolomics for human disease diagnostics, no diagnostic test based on metabolomics has yet reached the clinics [17]. There are several reasons and explanations for this observation, the most frequently cited being the difficulty to integrate multiscale biological information to generate knowledge and predictive models [13, 16], the inappropriate design of clinical trials with an often too small number of patients, together with a lack of validation cohorts [16, 18], but also due to issues at the level of data acquisition, i.e., analytical chemistry. Indeed, the lack of standardization of the data production methods, together with the expression of results in a semi-quantitative manner, are frequently highlighted as factors preventing the sharing and reuse of metabolomics data, and their integration into multi-omics models [16, 18]. Anyway, while a growing number of studies report on metabolic signatures for the diagnosis and monitoring of pathologies, responses or non-responses to treatments, it is now necessary to consider converting these complex and multiparametric signatures into reliable assays, with appropriate costs of sample analysis, that could be run in medical biology laboratories and even point-of-care tests, which are part of the participative dimension of personalized medicine. These aspects, which fully deal with analytical chemistry, are still poorly addressed within the metabolomics research community.

In this context, this review deals with the main challenges linked to analytical chemistry that need to be overcome to foster the implementation of metabolomics in personalized medicine and in clinical practice, from data production for biomarker discovery and validation, to the translation of metabolomics signatures into assays for medical laboratories and point-of-care tests.

## Metabolomics for personalized medicine: more standardized and sharable metabolomics datasets are still required

Several recent reviews have already addressed clinical applications of metabolomics in various fields such as oncology, cardiology, neurology, diabetes, kidney and liver diseases, and also response to treatments (aspirin, simvastatin, or antihypertensive drugs) [16, 18–21].

In their article entitled “Metabolomics for the masses: the future of metabolomics in a personalized world,” Trivedi et al. have discussed on the current positioning of metabolomics and on its future in a context of personalized medicine and, more generally speaking, healthcare. They have listed around one hundred metabolomics studies proposing biomarkers of various pathologies and published between 2000 and 2017. The authors pointed out limitations at different levels: data production with a lack of interoperability and methodological validation, poor experimental design with many studies dealing with less than one hundred subjects (leading to a lack of statistical robustness and validity), with the absence of replication/validation cohorts. Finally, stating that mass spectrometry–based approaches are not suitable for large-scale screening of a very large population due to their low throughput and high price, they consider alternative analytical chemistry tools accessible to non-specialist end users, such as lateral flow devices, dipstick approaches, or breath measurements of volatiles [18].

The same conclusions are presented in the position paper of Pinu et al., which summarizes the discussions on translational metabolomics undertaken during the peer session of the Australian and New Zealand Metabolomics conference in 2018. In addition to issues related to the lack of standardization of data acquisition protocols and to partial identification and too limited annotation of datasets, these authors also emphasized the insufficient recognition of metabolomics by funding agencies, the difficulty of gathering multiple fields of expertise within a given group, and the necessity to develop routine tests and portable devices [16].

**Fig. 1:**
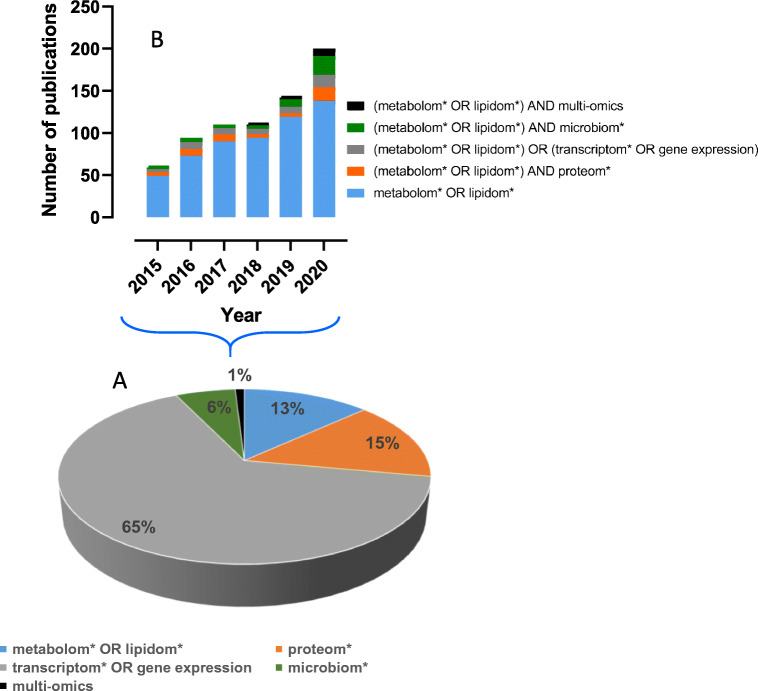
Omics technologies for biomarker discovery in the medical field. (A) Pie chart displaying the relative contributions of the various omics approaches for the discovery of biomarkers of diseases over the 2015–2020 period. The PubMed database was inquired (March 2021) with the following keywords occurring in titles and/or abstracts: “disease*,” *marker* or signature*, patients; and “metabolom* or lipidom*,” “transcriptom* or gene expression,” “proteom*,” “microbiom*” and “multi-omics,” excluding review articles. (B) Number of publications related to metabolomics and/or lipidomics alone or combined with other omics from 2015 to 2020

A search in the PubMed database (March 2021) over the 2015–2020 period with keywords related to the main omics techniques, combined with the terms “disease*,” “*marker* or signature*,” and “patients,” and restricted to the occurrence of these words in titles and/or abstracts, yielded 4874 publications. As shown in Fig. 1(A), the bulk of these studies involves transcriptomic analyses. Of these, the contribution of metabolomics or lipidomics analyses amounts to 13%, that of proteomic analyses to 15%, while approaches linked to microbiome and multi-omic analyses are emerging. Figure 1(B) displays the number of publications mentioning the use of metabolomics or lipidomics alone or with other omics approaches. A fourfold increase in the number of publications referring to metabolomics has been observed between 2015 and 2020. Although metabolomics analyses are still mainly used alone, there is a trend toward increased use of multi-omics approaches in 2020 (62 publications out of 187). Finally, among the 643 publications related to metabolomics or lipidomics, only 15 of them mention the use of a validation cohort to confirm the molecular signatures obtained.

At the same time, data repositories have been developed, making publicly available projects and studies including raw data together with analytical and biological metadata necessary for statistical analysis, as well as processed and annotated datasets. The two main data warehouses in the field of metabolomics are MetaboLights [22, 23] and Metabolomics Workbench [24]. They allow sharing of protocols, assays, and even tools to perform analyses and meta-analyses on the datasets (for Metabolomics Workbench). At the time of writing this review, MetaboLights (https://www.ebi.ac.uk/metabolights, accessed March 2021) contained 554 studies, of which 30% deal with human studies (i.e., related to “homo sapiens” in the browse study menu) and about 18% are performed on human blood and urine samples (i.e., related to “blood,” “serum,” “plasma,” “whole blood,” and “urine” in the browse study menu). Metabolomics Workbench (https://www.metabolomicsworkbench.org, accessed March 2021) included 1370 studies of which 41% dealt with human biological samples. A large number of projects are related to diseases such as cancer (182 projects) or diabetes (63 projects). It should also be noted that spectral data warehouses such as MassIVE (https://massive.ucsd.edu), a community resource developed by the NIH center for computational mass spectrometry for the free exchange of mass spectrometry data, are likely to host metabolomics datasets (404 out of 11,065, March 2021).

The development of data warehouses has been facilitated by the efforts made within the community to standardize the different steps of the metabolomics analysis workflow. Initially, the Metabolomics Standards Initiative, set up by the Metabolomics Society in 2005, has addressed, through working groups, various aspects related to ontology problems, data exchange, biological metadata, chemical analyses (from the reporting of analytical chemistry metadata to metabolite identification status), or data processing and statistical analyses [25]. Regarding raw data sharing, vendor independent standards have been proposed since the 1990s, and nowadays, XML (i.e., “eXtensible Markup Language”)–based formats such as mzML [26] and nmrML [27] are supported by vendors and commercial and open-source software [28]. Concerning metadata associated to studies, the ISA-Tab format (“ISA” for Investigation, Study, Assay, and “Tab” for tabular) has been developed to collect and share both analytical chemistry and biological metadata [29]. Finally, certified reference materials are provided by metrological institutions, which facilitate the implementation of international inter-laboratory studies [30]. This is, for example, the case for human plasma with the SRM 1950, which is produced by the U.S. National Institute of Standards and Technology (NIST) [31], and which has been used in several inter-laboratory tests [32–35]. Beyond the case of human blood samples, NIST urine reference materials have been used in an inter-laboratory study involving NMR, GC-MS, and LC-MS untargeted metabolomics analyses [36], and the development of a human stool reference material for metabolomics and metagenomics gut microbiome analysis is envisaged, as highlighted in a workshop report [37].

However, despite all these advances, the use and reuse of public data from global metabolomics analyses in the frame of meta-analyses remain uncommon. Table 1 shows the main meta-analyses published to date. They are all based on mass spectrometry data, and some of them use software tools such as MetaboAnalystR 3.0 [38] or PAIRUP-MS [39], which enable joint analyses of different projects or batches from raw data and pathway enrichments.

**Table 1:** Meta-analyses involving untargeted metabolomics-based approaches

Publication title	Data	Technology	Software	Reference
Comprehensive meta-analysis of COVID-19 global metabolomics datasets	7 datasets from 3 countries, including 5 raw datasets from MetaboLights, MassIVE, and authors, and 2 annotated peak tables from 2 publications. 438 blood samples from 337 subjects	LC/HRMS	MetaboAnalystR 3.0	Pang et al., *Metabolites*, 2021 [40]
Benford’s law and metabolomics: a tale of numbers and blood	Datasets from 3 studies performed by the author, no raw data available, peaktable available for one study	LC/HRMS	No	D'alessandro, *Transfus Apher Sci*, 2020 [194]
Integrating untargeted metabolomics, genetically informed causal inference, and pathway enrichment to define the obesity metabolome	3 LC/MS datasets, no raw data available, one peaktable available (related to the software publication)	LC/HRMS	PAIRUP-MS	Hsu et al., *Int J Obes (Lond)*, 2020 [195]
MicroRNAs regulating human and mouse naïve pluripotency	Meta-analysis including microRNA-seq, RNA-seq, and metabolomics datasets; the metabolomics datasets are from a single published study; peaktables available; no raw data available	LC/HRMS, LC/QQQ-MS, GC/MS	No	Wang et al., *Int J Mol Sci*, 2019 [196]

The study of Pang et al. deals with a comprehensive meta-analysis of COVID-19 global metabolomics datasets. It was achieved by using seven liquid chromatography coupled to mass spectrometry datasets obtained from six studies that were realized in three distinct countries. Five datasets were obtained as raw data from public repositories or from the authors of the studies, whereas the remaining two datasets consisted of annotated peak tables obtained from supplementary materials of publications. The authors implemented a computational workflow to process the raw data coming from different experiments and performed pathway enrichment and visual data mining, leading to metabolic signatures characteristic of the disease progression and clinical outcomes [40].

In summary, progresses have been achieved in terms of technological advances for the production and analysis of metabolomics data, and for the standardization and sharing of these data. These advances are reflected in the increasing involvement of metabolomics data in clinical studies dealing with disease biomarker discovery, and in systems medicine research projects based on multi-omics approaches. However, global and non-targeted metabolomics analyses are still very little used, or even not at all, in routine care practices, especially in medical laboratories. It is therefore necessary to pursue research activities in the field of data production and interoperability, as detailed below with a focus on mass spectrometry–based metabolomics approaches.

## Toward inter-operable and reusable metabolomics data for biomarker discovery: from appropriate project design and sample collection to confident identification and measurement of biomarker candidates

The pipeline of biomarker development includes several key stages, consisting of discovery, validation, and clinical translation [41]. Each of these steps has its own limitations and can be improved. For instance, the discovery phase might suffer from a lack of standardized and validated methods, yielding poor experimental reproducibility between laboratories. This is especially the case with MS-based metabolomics, whereas, although detecting less metabolites than LC-HRMS, NMR can be more directly quantitative and can thus deliver more robust data than untargeted LC-HRMS-based approaches. Another reason is that clinical studies are not always appropriately designed for biomarker discovery, with biomarker candidates not validated in independent cohorts and also not sufficient clinical phenotyping available [42, 43]. In this context, in the following paragraphs, we will review and discuss the recent findings in LC/MS-based metabolomics that can fill these gaps by pinpointing the need of the following:

- Large multi-center cohorts as well as validation cohorts to increase statistical power and biomarker specificity and avoid confounding factors
- Improved metabolome coverage and metabolite identification confidence level (e.g., thanks to optimized and validated acquisition workflows, dedicated spectral databases)
- Standardized data production workflow with improved robustness (e.g., with QCs, batch-to-batch consistency/normalization, large-scale quantification) and capability of automated interpretation of the huge amount of data generated
- Linked untargeted and targeted quantitative approaches for proper analytical validation of biomarker candidates

### The issue of design of experiment regarding cohort samples: toward more standardization

**Fig. 2:**
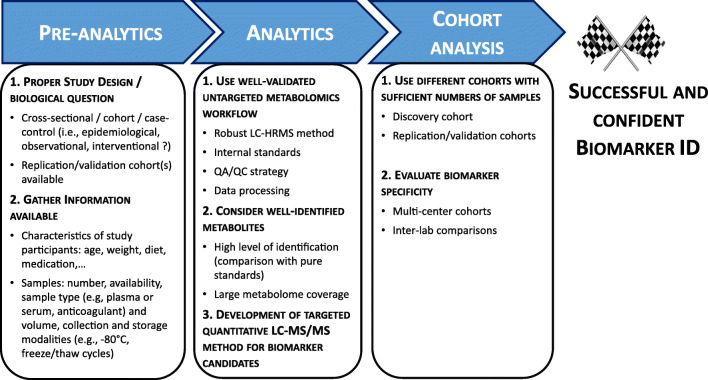
The road to successful biomarker discovery

Metabolomics is a question-driven method. Thus, the prerequisite of a successful metabolomics experiment is a well-defined clinical question, which would unarguably imply active discussions between physicians, analytical chemists, and data scientists, each one knowing the constraints and requirements of their respective field. A successful metabolomics study requires some key elements, including but not limited to (i) consistency of samples to be analyzed and compared (e.g., individuals matched for sex, age, weight, ethnicity, lifestyle factors, etc.; site and type of sample, sample shipment, storage, and handling), (ii) proper study design, (iii) proper control groups and conditions, and (iv) sufficient sample size of compared groups to encompass inter-individual variability and provide statistical power [44] (Fig. 2).

#### Preanalytical considerations, study design

The levels of metabolites constituting the metabolome of any individual are quantitatively affected by many factors such as disease, drug use, environmental exposures, or nutrition [6, 45]. Therefore, collection of data related to demographic, lifestyle, and physiological factors (e.g., diet, gender, ethnicity, age, and BMI) is necessary to limit inter- and intra-individual variations and identify potential confounding factors. This practice also ensures that appropriate data can be incorporated into the experimental design and data analysis processes [46].

Obviously, sample type/matrix must be consistent between the patient samples and the control group (e.g., plasma EDTA). Although overall concordance in results and similar discriminatory ability can be observed when running plasma and serum metabolomics [47], serum appeared to show higher levels of some metabolites as a potential consequence of the clotting process, as it is exemplified in the study of Wedge et al., which deals with the metabolomics analysis of 29 small-cell lung cancer patients [48]. Some metabolites can also readily degrade or interconvert during sample preparation or during temporary storage at room temperature before analysis [49–51]. Kamlage et al. reported on the impact of blood and plasma processing on the concentration of metabolites. They observed significant metabolite concentration variations (in the 4–19% range) in the case of hemolysis or short-term storage of plasma samples at room temperature or cooled on wet ice, and also some minor consequences when samples were contaminated with buffy layer or in case of micro-clotting [52]. Caution has to be paid to avoid these issues.

Different study designs (e.g., cross-sectional, cohort, case-control studies) have their own strengths and weaknesses, and their applicability depends on the clinical question of interest [53]. Moreover, the sample size of groups should be similar and large enough in a comparison to provide sufficient statistical power. However, it is important to note that some cohort studies may use more controls than cases in their experimental design, which might imply the development and use of specific statistical tools for the treatment of the resulting metabolomics data. In the case of expected high inter-individual variability, a larger sample cohort would even be required. The issue of statistical power related to sample size for the appropriate design of metabolomics studies has already been addressed through modeling approaches using pilot studies [54], or even when pilot studies are not available [55]. However, there is still a lack of reference procedures to address this point [56]. Thus, experts in data production and statistical analysis should be involved in experimental design, together with the sponsors of the study [57–59]. They should pool their expertise to ensure the most standardized and robust experimental design. Important points to be discussed and agreed are the following: (i) a clear definition of the research question, an as-precise-as-possible description of clinical characteristics of each group to be compared (in order to avoid any unwanted inter-group variability); (ii) a “true control” group (although it is often complicated to collect samples from healthy people in hospitals); (iii) an estimation of the minimal sample size that is required to address the biological question, the number of sample aliquots needed for the study; and (iv) the creation and sharing of a single exhaustive file of sample metadata with all known technical and clinical variables for each sample (sample name, tube labeling or barcode, sample box, location in the box, collection origin, collection date, gender, age, clinical treatment, diet, disease, BMI, used anticoagulant, ethnicity...). Such a file could help to better define the experimental groups in terms of homogeneity, while avoiding any confusion and limiting the occurrence of possible confounding effects (Fig. 2).

#### Discovery and (pre)validation cohorts

As mentioned above, untargeted LC-HRMS metabolomics methods are mainly used for biomarker discovery. The resulting biomarker candidate data should be considered preliminary until their validation on a larger cohort with the use of a similar untargeted workflow or a targeted quantitative metabolomics approach. Indeed, a common issue in metabolomics-based biomarker discovery is the absence of replication and insufficient sampling, with many studies involving a single cohort of limited size, i.e., often fewer than 100 samples [60]. Replication of the results on biomarker performance using independent multi-center cohorts is needed to ensure proper biomarker validation for further transfer to the clinics [18]. Collaborative networks and access to well-characterized bio-banked samples are often needed to perform such large-scale studies.

#### Standardized sample preparation

As the level of comparability/variability is the critical point to be controlled for standardizing metabolite concentration in the frame of untargeted metabolomics experiments, standard operating procedures (SOP) for sample collection and metabolite extraction are essential [57, 61, 62]. The review of Kirwan et al. [57] gives a valuable overview about well-defined and validated protocols for the collection of samples for metabolomics research.

Kirwan et al. listed several already published protocols for collection and storage of numerous biofluids and tissues. Important insights are synthesized in an informative table highlighting the crucial points of the protocols related to metabolomics: (i) collection methods, (ii) range of temperature before processing, (iii) storage conditions and reported consequences if not followed, and (iv) observed confounding effects [57].

Before implementing an extraction protocol chosen as the most appropriate to address the biological question of interest, a crucial step in the standardization process consists in sample normalization in terms of metabolite concentration before data acquisition [62]. Regarding serum and plasma analyses, a consensus normalization way is to analyze the sample using a fixed sample volume [61, 63]. As for a biomedical analysis, the sample must be collected in a fasting state to minimize unwanted sources of variability on the metabolome. For human fecal samples, Karu et al. also described several proposed protocols for human fecal metabolomics [64]. We advise to normalize on the dry weight of the freeze-dried stool sample to avoid variability induced by differences in water content from one fresh stool sample to the other [65]. Regarding urine metabolomics studies, the need of normalization due to diuresis variations is well known and it has been already addressed in several publications. Ideally, all urine samples in a study should be collected over a period of time such as 24 h, knowing that the metabolic content of the sample can be impacted by bacterial growth and chemical stability issues [50]. The most used normalization protocols rely on creatinine, osmolality, total useful signal (TUS, post-acquisition normalization), and specific gravity measurements [66–71]. Although no clear recommendation has emerged, the limitation of using creatinine, encountered in many pathological contexts, has been highlighted many times, while osmolality and specific gravity appear to be the most reliable normalization protocols [68, 71, 72].

For tissues, it is often difficult to weigh accurately a small piece of frozen sample. We advise a standardization related to the measurement of the total protein concentration. The protein concentration is thus measured in the pellet obtained after the protein precipitation step during the metabolite extraction (e.g., by using bicinchoninic acid “BCA” protein assay). While for bacteria the normalization can be made based on the optical density measurement [73, 74], normalization of eukaryote cell samples before acquisition can be done on cell numeration (which may lack of accuracy), or more confidently by using total protein concentration or DNA concentration in the extract. To conclude, it is essential to normalize the concentration of metabolites present in the final extract and before acquisition to detect only the metabolic variations related to the clinical parameter being monitored. Post-acquisition normalization can also be implemented, for example, by using the TUS (as mentioned above for normalization of urine samples) [68].

### The issue of metabolome coverage and accurate measurement

Obtaining an exhaustive picture of the metabolome is highly desirable to increase the likelihood of getting the best biomarker or set of biomarkers. Although major developments have already taken place, we are still a long way from getting a comprehensive coverage of all the metabolites. In the absence of a universally accepted procedure for biomarker discovery by mass spectrometry–based metabolomics, each data production facility uses its own optimized procedure [18]. To achieve broader metabolite coverage, samples are often analyzed several times by complementary LC-HRMS(/MS) methods [63, 75–77]. A large portion of the detected signals remains, however, structurally uncharacterized, and thus, metabolite identification still represents a major bottleneck in metabolomics [4, 78]. Detecting as many metabolites or metabolite features as possible with the objective of obtaining maximal biochemical information is a general tendency. However, confidently identifying and measuring true and unique metabolites is a completely different objective that is absolutely required to obtain both reliable biomarker candidates and meaningful biological information readily sharable between laboratories. Therefore, broad metabolome coverage makes sense only if metabolites are annotated/identified at a high confidence level. Of course, this might sound frustrating or can be erroneously linked to poor methodological performances since this often yields limited sets of metabolites (e.g., ~ 200), instead of few thousands of metabolite features or elemental compositions. Such “limited” number of metabolites can already provide key insights into relevant clinical questions. For example, the robust monitoring of 137 metabolites in the blood of 800+ patients with acute decompensation of cirrhosis with/without acute-on-chronic liver failure (ACLF) provided unprecedented insights into the biochemical mechanisms, underlying the development of the ACLF syndrome and also a 38-metabolite blood fingerprint specific for ACLF that revealed mitochondrial dysfunction in peripheral organs [79, 80].

#### Confident annotation and identification of metabolites

As mentioned above, accurate identification and monitoring of metabolites are prerequisites to achieve measurements’ reproducibility across laboratories and among countries. Confident metabolite identification in complex biological matrices requires the combination of several information lines exploited in conjunction. High mass resolution (> 100,000, *M*/Δ*M*, full width at half maximum) and high mass measurement accuracy (< 1 ppm) allow for the measurement of isotope pattern and isotope fine structure, including the distinction of isobaric isotopes [81–83]. Complementarily, accurate retention time(s) and MS/MS spectra can provide high confidence in metabolite identification when matched to reference data included in chemical/spectral libraries. Among those complementary information lines, one of the most valuable elements to confirm metabolite annotation or reduce the list of possible annotations is the acquisition of fragmentation spectra and their comparison to reference MS/MS spectra included in reference mass spectral libraries [82]. Differently from proteins, fragmentation of metabolites under low-energy conditions is relatively unpredictable (at least with a high confidence level). Therefore, the most relevant mass spectral databases for definitive identification of metabolite biomarker candidates are those obtained from pure authentic standards, and are thus unfortunately limited by their (most often commercial) availability.

#### Databases are essential but often reflect only what is identified and commercially available

**Fig. 3:**
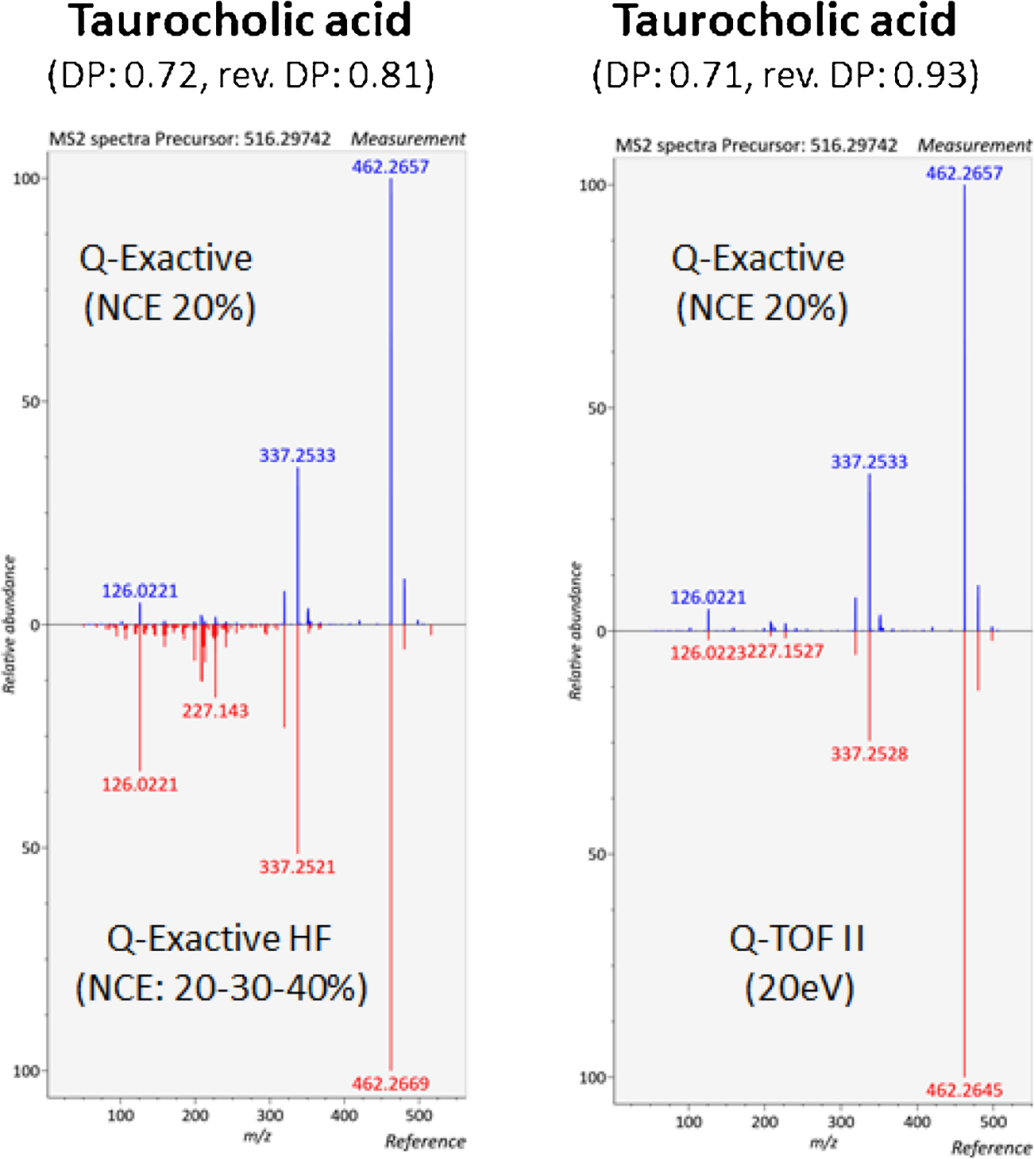
Head-to-tail comparison of evaluated versus reference MS/MS spectra of taurocholic acid obtained under non-resonant conditions. Evaluated MS/MS spectra (blue color) were obtained in the positive ion mode on a Thermo Q-Exactive instrument (NCE 20%). Reference spectra (red color) are stored in the MoNA database and were recorded in the positive ion mode on a Q-Exactive HF (NCE 20–30–40%) and a Waters Q-TOF II instrument (20 eV). [M+H]+ ion at *m*/*z* 516.2974. Spectral matching was performed using the MS-DIAL version 4.12) software [208]. DP, dot product; rev. DP, reverse dot product

Most widely used (public and proprietary) spectral databases include MassBank [84], HMDB [85], GNPS [86], MoNA (https://mona.fiehnlab.ucdavis.edu/), LIPID MAPS [87], NIST 20 (https://chemdata.nist.gov/dokuwiki/doku.php?id = chemdata:msms), METLIN [88], and mzCloud (https://www.mzcloud.org/). For a more detailed comparison and discussion of these databases, we recommend these articles in the field [89, 90]. However, in brief, procedures for data collection and curation, instruments used (e.g., Orbitrap, Q-TOF), fragmentation conditions (e.g., resonant and/or non-resonant conditions, number of collision energies, MS^n^), and type of molecules differ from one database to the other. Presently available spectral databases are therefore not strongly overlapping [90], which underlines their complementarity as exploited by recent studies for larger metabolite identification [91]. Interestingly, a European proposal for quality control and quality assurance of tandem mass spectral libraries has been recently published and also reported that Q-TOF and Orbitrap-based instruments yielded comparable MS/MS spectra [92]. As a representative example, the MS/MS spectrum of taurocholic acid as acquired in the positive ion mode on a first-generation Thermo Q-Exactive instrument (at a normalized collision energy “NCE” of 20%) has been successfully matched to spectra stored in the MoNA database and previously recorded both on a Q-Exactive HF and a Waters Q-TOF II instruments used under different fragmentation conditions (Fig. 3). This opens the door to spectral databases generated in the FAIR (i.e., “Findable, Accessible, Interoperable, Reusable”) data context [93] also with a standardized way of describing the observed ions [94].

Although covering more and more metabolites, not all metabolites found naturally in biological matrices are currently commercially available to feed those databases, which clearly limits their expansion. As a matter of fact, metabolomics researchers usually focus on metabolites that are present in databases or websites. Although very exciting for the analytical chemist, the exploration of unknown metabolites, the so-called dark matter [95], is one of the biggest challenges and a research field on its own and is therefore still insufficiently explored. Therefore, acquisition and thorough annotation (or putative identification) of MS/MS data from biologically relevant and recurrently observed metabolite features absent from databases and chemical provider catalogs is required for their further implementation and storage in specific shared databases. Such a strategy has been recently used to build spectral libraries of unidentified but annotated recurrent spectra derived from NIST urine samples [96], and could be extended to unknown signals returned by meta-analysis at the pathway level with software tools such as Mummichog [97].

### Reporting high-quality and robust data in the frame of untargeted MS-based metabolomics studies

#### General quality assurance and quality control practices

Quality assurance (QA) and quality control (QC) are indispensable processes in research and critical in metabolomics to obtain high-quality and FAIR data [58, 59, 98, 99].

QA deals with processes planned and performed before sample collection to fulfill predefined quality requirement. The main processes for metabolomics experiments are staff training, preventive instrument maintenance, analytical methods validation, and calibration of analytical tools and verification of their performance.

Quality control deals with techniques and activities used to measure and report if these QA requirements have been met during and after data acquisition. It can include (i) run order randomization to control unwanted variation and unwanted correlation; (ii) internal standards added to samples in order to report the quality of data within and between batches; (iii) blank samples to assess the occurrence of contaminations, interferences, artifact feature, carry-over phenomenon, and matrix effect (if internal standards are added); and (iv) QC samples (including diluted QC in order to highlight artifactual features).

QC samples should be representative of the qualitative and quantitative composition of the samples to be analyzed. The samples can be obtained by pooling aliquots of each original sample and then following the same sample preparation protocol of the biological samples. Proposed in 2006 for the first time for untargeted metabolomics studies in order to check for data quality [100], QC samples have been and are used in numerous studies and several publications have highlighted their importance in the metabolomics workflow [59, 61, 98, 99, 101]. Broadhurst et al. have reported on the different kinds of QC samples, and their respective advantages and limitations [98]. QC samples are usually injected several times at the beginning of each batch analysis in order to equilibrate the analytical platform, and then every 5 to 10 biological samples in order to control the stability and performance of the analytical platform, and its reliability during the course of analysis. They have also a crucial role in data pre-processing, as emphasized below.

#### The need for reproducible data (pre)processing workflows and precisely documented metabolite identification procedures

This includes data preprocessing and processing, the reporting of metabolite identification, and publication and data sharing.

The data-preprocessing step deals with the implementation of a peak-picking (i.e., detection and integration of LC/MS peaks, i.e., features), feature alignment and feature integration workflow. This is a key step in untargeted metabolomics, which leads to data matrices that have to be cleaned and annotated before being subjected to statistical analyses. Software packages dedicated to NMR, GC-MS, or LC-HRMS are available for that, and their description is beyond the scope of this review. There is a need to standardize these pre-processing steps [102], but most importantly to standardize the sharing of processed data so that they can be re-analyzed by other investigators to advance in the development of these tools. This can be achieved by integrated software tools that combine several data treatment steps, from data preprocessing to statistical analyses, such as workflow4metabolomics [103] or Metaboanalyst [104], the latter also proposing some data visualization and metabolic pathway enrichment tools. Such metabolomics data-processing platforms enable to store and share all the parameter values related to data pre-processing and processing.

Dataset annotation and metabolite identification are crucial steps enabling to convert hundreds to thousands of metabolites features contained in peak tables into biologically interpretable data. The way of reporting metabolite identification has already been highlighted as a key issue. The Chemical Analysis Working Group of the Metabolomics Standards Initiative has thus proposed four levels of confidence for metabolite identification [105]:

- Level 1: fully identified compounds based on at least 2 orthogonal (i.e., independent) data related to an authentic standard analyzed in the same experimental conditions.
- Level 2: putatively annotated compounds based on characteristic physicochemical properties or spectral similarity with spectral libraries. In this case, there is one candidate, but the authentic standard is not available for confirmation.
- Level 3: putative characterized compound classes, based on characteristic physicochemical properties of a chemical class (i.e., acylcarnitine derivatives, or sulfoconjugates). In this case, several candidates are possible and it is not possible to highlight a precise chemical structure.
- Level 4: unknown compounds.

Although they are simple to implement, these identification levels have limitations since they cannot avoid ambiguities. For example, the use of at least two orthogonal data together with that of a standard, as it is required for a level 1 metabolite identification, does not always lead to an unambiguous identification, as observed with optical isomers such as enantiomers, for example [106]. Although improvements have been proposed with the addition of a fifth level [107], or with the proposal of metabolite identification metrics with a quantitative scoring system based on the sum of all the data types supporting the identification [108], there is a need to propose a metabolite identification reporting system that focuses further on chemical structures.

### Application to different types of medical cohorts

Depending on the level of complexity of the study, different kinds of quality control procedures should be applied to the metabolomics data production workflow in order to achieve a sufficient degree of standardization. Thus, non-targeted metabolomics studies performed with MS technologies can be classified into three categories:

- (a) Studies dealing with a sample set analyzed in a single batch, in the same laboratory, and with the same instrument

- (b) Studies dealing with a large cohort requiring to be analyzed in several batches, over a long period of time, in the same laboratory

- (c) Studies dealing with a large cohort and requiring several laboratories

All the QA and QC procedures previously detailed below apply in terms of experimental design, sample preparation, data production, and pre-processing. The most important element is to use QC samples and to analyze them at regular intervals in order to guarantee signal stability over the duration of the experiment.

#### Studies dealing with a sample set analyzed in a single batch, in the same laboratory and with the same instrument

This case deals with cohorts ranging from 200 to 500 samples, depending on the kind of sample preparation protocols and instruments used. It is the simplest one because recommendations on how to standardize the different steps of the untargeted metabolomics workflow and quality management practices (from the experimental design of the study to the submission of data into public repositories) are available and have been the subject of many publications [30, 57–59, 61, 62, 98, 99, 105, 109–113] (Table 2).

**Table 2:** Selected publications dealing with recommendations and guidelines regarding metabolomics workflows

**References**	**Experimental design**	**Sample collection**	**Sample preparation**	**Data acquisition**	**Data pre-processing**	**Data processing**	**Metabolite identification**
Sumner et al., 2007 [105]	–	–	Protocol and extraction methods	Instrumental conditions and performance, method validation	Peak detection/integration	–	Metabolite identification
Goodacre et al., 2007 [197]	–	–	–	–	Peak detection/integration	Data mining, statistical analyses	–
Dunn et al., 2011 [61]	QA/QC, large cohorts	Large-scale studies	Serum, plasma	GC and LC-MS, samples, and pooled QC	Data preprocessing workflow	Data processing workflow	Levels of confidence, unknown metabolites
Dudzik et al., 2018 [59]	QA	Plasma, serum, urine, cells, tissues	Plasma, serum, urine cells, tissues	Instrumental conditions, batch and matrix effects, carryover	QA/QC	QA/QC	–
Kirwan et al., 2018 [57]	Project planning	Plasma, serum, urine, feces, saliva, CSF, tissues	–	–	–	–	–
Broadhurst et al., 2018 [98]	–	–	Pooled-QC preparation	Pooled-QC: precision, conditioning	–	Pooled-QC: inter batch correction	–
González-Riano et al., 2020 [62]	–	–	Plasma, serum, urine, feces, cells, tissues	Multi-targeted metabolomics, GC-MS, CE-MS, IMS, chiral analysis	Peak detection/integration	Data cleaning normalization, confounding factors, variable selection	GC-EI-MS (commercial or in-house spectral libraries), LC-MS and CE-MS solutions
Rampler et al., 2021 [30]	–	–	Discussion on protocols	Absolute quantification, reference material	Peak detection /integration	MS-based multi-omics, merging metabolomics and lipidomics	Metabolite and lipid annotation

#### Studies dealing with large cohorts requiring to be analyzed in several batches, over a long period of time, in the same laboratory

In this case, in addition to the already described QA and QC procedures, it is important to have a “long-term reference (LTR)” QC sample, as proposed by Dunn et al. [61]. In some cases, the strategy of the “pooled QC sample” might not be the most relevant. For instance, when analyzing some particularly large cohorts, the amount of QC sample available can become limiting; while for projects involving rare diseases or longitudinal studies, the exact number of batches to be received can be unknown or the samples are not all available at the beginning of sample preparation and analysis. In such situations, a commercially available plasma LTR QC sample from NIST (see below) can represent a reliable alternative.

There are several key issues associated with these LTR QC samples. The first one deals with how representative such samples are, in terms of metabolites and concentration ranges. As an example, the standard reference material (SRM) dedicated to metabolites in human plasma (SRM 1950) is prepared from 100 donors, with an equal number of men and women of 40 to 50 years of age, selected to be representative of the ethnic distribution of the US population [31]. However, it is not established whether or not this SRM will be appropriate for studies performed on other populations, or on patients affected by overt diseases or metabolic disorders. Further studies are needed to address these issues.

The second one is their long-term stability. Indeed, stability studies are challenging to implement in the case of non-targeted and semi-quantitative approaches, especially those based on the use of mass spectrometry, due to the impossibility to ensure the stability of the metabolites contained in the QC samples that are used for inter-batch normalization. However, a few studies dealing with untargeted metabolomics approaches have been published on this issue. For example, Laparre et al. have evaluated the impact of the storage temperature (+ 4 °C, − 20 °C, − 80 °C, and freeze-dried stored at − 80 °C) and the storage duration (5 to 144 days) on the bovine urinary metabolome by using liquid chromatography coupled to high-resolution mass spectrometry [114]. The authors focused on 200 identified metabolites contained in their spectral database. Normalization was performed by dividing each feature’s intensity recorded on the different days by the corresponding feature’s intensity recorded in the freshly collected urine sample at day 0 for every subject. Furthermore, scaling factors were calculated by dividing the average intensities recorded in − 80 °C stored samples per feature and for each batch by the corresponding intensities recorded in the reference batch. By these means, they found that urine metabolic profiles are altered starting from 5 days when stored at + 4 °C, and after one month at − 20 °C. The temperature of − 80 °C was considered as the most convenient urine long-term storage condition. In another study, Palmer et al. have investigated the 12-month stability of dried blood spots (DBS) and dried urine spots (DUS) at different storage temperatures (− 20, + 4, and + 21 °C) and compared it to plasma and urine biofluids stored at the same storage temperatures and time by using LC/HRMS-based untargeted metabolomics [115]. Inter-batch normalization was achieved using a pooled QC sample. They concluded that DBS and DUS stored at + 21 °C are stable for up to 4 weeks but are not stable over a 1-year period, whereas they showed good stability when stored at − 20 °C for 1 year.

These two studies are based on the assumption that QC samples used for inter-batch normalization are stable in “reference storage conditions” (i.e., at − 80 °C or − 20 °C). However, some studies using absolute quantification and/or a calibration system highlighted some altered metabolite concentrations in plasma samples stored at − 80 °C up to five years. By using the Biocrates AbsoluteIDQ p180 targeted-metabolomics assay, Haid et al. observed significantly changed levels of amino acids, acylcarnitines, glycerophospholipids, sphingomyelins, and the sum of hexoses, with average increases or decreases of + 13.7% or − 14.5%, respectively [116]. Otherwise, Wagner-Golbs et al. analyzed EDTA plasma samples stored for up to 16 years by gas and liquid chromatography-tandem mass spectrometry-based quantitative metabolomics. They found that 226 out of 231 metabolites remained stable during the first seven years of storage [117].

There is a limited number of large-scale studies using untargeted metabolomics that have been published. Among those, a representative one is that of Dunn et al. who have reported on the molecular phenotyping of 1200 “healthy adults” from the UK in the age range of 19–81 years, by using GC/MS and LC/HRMS-based metabolomics [118]. The data were acquired across 11 months in 10 batches including samples from 120 subjects analyzed within a five-day period. Data were processed using dedicated GC- and LC-HRMS workflows, and inter-batch normalization was achieved thanks to a unique QC sample and the LOESS algorithm. From 259, 7813, and 7914 metabolite features initially detected in GC-MS, LC-HRMS (positive mode), and LC-HRMS (negative mode), the implementation of signal correction, batch integration, and quality assurance procedures led to 126, 2181, and 2283 metabolite features combined within a single multi-batch data matrix and available for statistical analyses.

Another interesting study is that of Sindelar et al. on the use of metabolomics to highlight prognostic markers of COVID-19 severity [119]. Seven hundred and four human plasma samples were collected at six-month longitudinal points from 341 patients, and SRM 1950 was used as QC sample. Given that the metabolic profiles were acquired over several months, the combined data showed strong batch effects that proved efficiently corrected by combined batch correction [120].

Chromatographic retention time shifts are the main issues regarding batch fusion over long periods. Particular software solutions have been described to correct within- and between-batch variability drifts in terms of mass accuracy, intensity, and retention times [121, 122]. Nevertheless, if there are too large differences in retention times between batches, it can become difficult to correctly align peaks with common automatic signal detection and alignment software tools [123, 124]. Then, targeted detection of metabolites that are present in laboratory spectral databases can be carried out using peak integration software from instrument suppliers. The batch fusion process would then be carried out more easily on the basis of known metabolites (targeted data treatment) and no longer based on *m*/*z* ratios and retention times alone and in a more blinded way (untargeted data treatment).

#### Studies dealing with large cohorts and requiring several laboratories

Although several laboratories with complementary technological expertise may be required in order to achieve the largest metabolome coverage, most studies dealing with a multi-platform approach are actually multi-omic studies involving a single metabolomics platform. In this case, generally, each type of omics data is analyzed separately in order to achieve molecular signatures, which are in turn collated and integrated by using molecular network analysis and visualization software tools [125].

Most of published metabolomics studies involving several laboratories or platforms with inter-laboratory comparison studies are dedicated to the evaluation of the performance and comparability of analytical methods [126–128].

The metabo-ring initiative gathered 5 NMR and 11 different LC/HRMS platforms with the objective of assessing the reliability of untargeted metabolomics approaches in obtaining comparable metabolomics profiles. Biological samples obtained from 2 different conditions were analyzed by the partners using their own in-house protocols. It was observed that, despite large differences in the number of spectral features produced after post-processing and the heterogeneity of the analytical conditions and the data treatment, the spectral information within and across technologies (NMR vs. LCMS) was highly convergent regarding 2 test datasets in terms of statistical analysis [126].

Izumi et al. performed an inter-laboratory comparison study on cell line extracts including 12 participating laboratories using their own analytical methods (capillary electrophoresis coupled to mass spectrometry, GC/MS and LC/MS with different kinds of high-resolution mass spectrometers and chromatographic conditions, corresponding to 15 and 9 methods for hydrophilic and lipophilic compounds, respectively). The aim of the study was to evaluate issues in integrating different kinds of metabolomics data. Overall, 203 metabolites and 580 lipid species were detected by at least one analytical method, among which 148 hydrophilic metabolites and 285 hydrophobic metabolites were detected by at least two methods [127].

Finally, in their article, Yu et al. describe the Consortium of Metabolomics Studies, which was established in 2014 for fostering large-scale collaborative research on medical cohorts and epidemiology. This initiative includes 47 cohorts from Asia, Europe, North America, and South America, and blood samples were analyzed by 17 platforms. It appeared, from 2 feasibility studies, that the overlap between any 2 different laboratories in terms of detected metabolites ranged from 6 to 121 metabolites within 5 leading laboratories, and that the median Spearman correlation coefficient was of 0.79 on 111 metabolites detected by two platforms. Absolute concentrations were provided on only 31 metabolites across the 5 platforms, and 28% of identified metabolites were not listed in public databases [128].

All these studies highlighted a modest overlap in terms of metabolite detection from one facility to the other, and two of them reported on inconsistencies in compound identifiers that limit the integration of datasets [127, 128]. This is especially the case with isomers that can be discriminated or not from one platform to the other. Such issues could be addressed by the development and sharing of spectral databases [33], by fostering the evaluation and the use of standard reference materials within metabolomics data production facilities [127, 128], and of course by providing absolute concentrations on identified metabolites present in the chemical libraries of data production facilities, in the frame of untargeted approaches.

### Analytical validation of biomarker candidates highlighted by untargeted metabolomics: the need for targeted quantitative metabolomics approaches

As mentioned above, confident metabolite identification is required for biomarker discovery. As a corollary, quantitative information about measured metabolites instead of relative differences must be provided if the biomarkers are to be used in clinical settings for diagnostic purposes through the definition of normal ranges of metabolite concentration. In addition to providing relevant data for answering clinical questions, metabolite concentrations expressed as molarity units will also facilitate access to the dynamics of the metabolome and of course the integration of metabolomics data with those of other omics, while also enabling facile comparison of results among laboratories and studies.

**Fig. 4:**
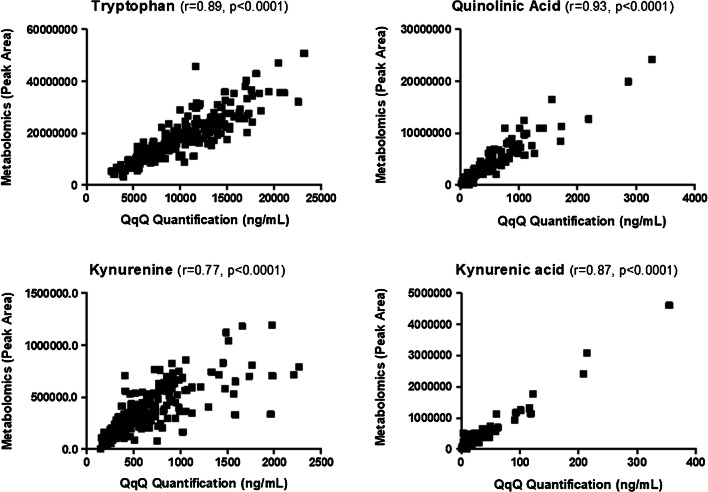
Comparison of metabolite concentrations measured by using untargeted and targeted approaches. Correlation between LC-HRMS data (peak area, exactive instrument) and absolute quantification data (ng/mL, Waters Xevo TQ-XS instrument) obtained for tryptophan, quinolinic acid, kynurenine, and kynurenic acid measured in the serum of 217 patients with different levels of cirrhosis decompensation. Correlation analyses were achieved by calculating Pearson correlation coefficients (*r*). Experimental conditions are displayed in the publication of Claria et al. [130]

Accurate and validated metabolite quantification is generally accomplished by using targeted LC-MS/MS-based approaches using low-resolution triple quadrupole instruments operating in the multiple reaction monitoring mode (MRM) and using metabolites labeled with stable isotopes (e.g., ^13^C, ^15^N, ^2^H) [129]. Such an approach can also be used to validate the results obtained by untargeted LC-HRMS metabolomics. For example, the concentrations of 4 tryptophan metabolites have been recently measured by LC-MS/MS (multiple reaction monitoring—MRM—mode with isotope dilution) in the serum of 218 patients with acute decompensation and ACLF in cirrhosis, and demonstrated excellent correlation with the corresponding MS signals observed under LC-HRMS conditions [130] (Fig. 4).

Although targeted LC-MS/MS (MRM) methods offer the best quantification sensitivity, they are often focused on a small set of compounds and thus suffer from such a limited metabolite coverage. New MS/MS workflows as implemented on high-resolution mass spectrometers such as Q-TOF or Orbitrap-based instruments have merged as powerful alternative strategies [131]. Such acquisition methods referred to as data independent acquisition (DIA) including the SWATH (accounting for Sequential Window Acquisition of all Theoretical fragment-ion spectra) approach are being increasingly used in metabolomics by enabling simultaneous metabolite identification and quantification through the acquisition of MS/MS spectra for all analytes in a single run [131, 132]. Although slightly less sensitive than traditional LC-HRMS or MRM approaches, recent publications highlighted the potential of SWATH approaches for unambiguous compound detection and accurate quantification in complex samples [133–136]. With constant instrumental improvements (e.g., data acquisition speed on Orbitrap mass analyzers, in collision energies management, sensitivity, MS^2^ spectra deconvolution), one can imagine great potential of such approach for future biological metabolomics applications. As a sort of proof of principle, Zha et al. recently reported on an innovative SWATH-to-MRM approach. Thus, a high-coverage targeted metabolomics method with 1303 metabolites in one injection was developed to profile colorectal tissues [137]. The success of such an approach would make LC-HRMS-based metabolomics both a screening and quantitative confirmatory technology.

## How to transfer metabolomics signatures from the research laboratories to the field

Metabolomics signatures are generally defined by clinical research studies, which cannot be considered as routine care practice. They are complex for several reasons. First, they are multi-parametric in that they often consist of few tens of metabolites. They often deal with small concentration variations from one group to the others, with concentration ratios often less than a factor five. Some of the metabolites of interest may be only partially characterized (i.e., for example a compound class, such as an acylcarnitine species including a hydroxylated carboxylic acid in which the position of the hydroxyl group cannot be precisely located). Furthermore, when obtained by using untargeted approaches, results are not expressed in molarity units but rather as peak areas, which obviously limits data reuse and sharing. Finally, these molecular signatures are often not validated due to a lack of validation cohorts, and also of specificity studies which are time consuming, expensive, and difficult to design.

Consequently, metabolomics signatures obtained from untargeted metabolomics cannot be directly used in the routine care practice. There is a need for simplification and for moving to quantitative results. Simplification can be achieved through statistical analysis tools enabling to select a small number of key components of the signatures. This is, for example, the case with the *biosigner* algorithm, which enables to find the smallest feature subset which significantly contributes to the performance of a multivariate statistical analysis model [138]. Regarding quantification, multiplexed targeted assays can be developed and implemented for specifically monitoring essential metabolites obtained from complex molecular signatures in order to confirm results obtained from untargeted metabolomics experiments, as already described in the previous section.

Furthermore, many actors and structures are involved in healthcare systems: clinical units in hospitals, medical laboratories in hospital settings or outside the hospitals, physician’s offices, pharmacists, and at least the patient at home. Current limitations in terms of data reuse and interoperability combined with a lack of validation data for the molecular signatures generated make metabolomics not yet easily usable in routine care. However, the main users in the short term could be clinical biologists and chemists, as well as non-experts, working in medical laboratories in a hospital context, near the clinical units. It is likely that in the near future the use of MS-based approaches will remain limited to hospital settings, operated by trained staff. This because (i) the sample preparation requires technically advanced operations and (ii) outputs of metabolomics workflows are complex molecular signatures of few tens of metabolites, often with small concentration variations. In the field of personalized medicine, the challenges will then lie in the longer term in the development and use of field rapid diagnostic tests based on the development of biosensors for the multiplexed and quantitative detection of several biomarker candidates.

### Metabolomics at medical laboratories

The clinical context has a strong impact on the manner of translating metabolomics signatures into clinical practice. It can be intended for a critical care, a chronic disease follow-up, or a genetic rare disease. Modalities such as the frequency of analysis, the delivery time of the results, the geographical availability (local, regional) of equipment, and the routine workflow have to be defined by taking into account this context. These aspects are usually managed in a clinical laboratory by a clinical biologist. One role of the biologist is to integrate these requirements with analytical constraints, by setting a framework in which the sample pathway, the analytical quality, and the delivery of the results are well defined.

Delivering a metabolic signature to a physician is challenging. This means to translate complex and numerous data into a self-explanatory analysis report, which must be available and interpretable for any clinician specialized in a given medical area. This translation requires processing, integration, and interpretation of data, in order to transmit a suitable information to the clinical issue. Clinical biologists will have a key role in the results transmission by integrating the biological message into the global clinical context. This requires rethinking medical biology by no longer reasoning in terms of isolated markers reflecting an organic function, but rather in terms of molecular signatures reflecting the stage of a disease at a given time. This is a major paradigm shift from a focus on broad categories of disease, to a more holistic approach that will integrate a patient’s metabolic status, impacted by all of their co-morbidities and their environment. To achieve this, clinical biologists will have to upgrade analytical technologies to generate data, integrate bioinformatics solutions, and develop an automated algorithm to express targeted and accurate results from complex data. Actually, clinical biochemistry is likely to undergo the same technological revolution as molecular genetics has undergone over the last 20 years with the advent of genomics and next-generation sequencing. One of the main challenges will be to obtain concentrations of key metabolites expressed in units of molarity, in order to meet the short-term needs of clinicians, while at the same time having the possibility of building databases of metabolic profiles that will be interrogated in different medical contexts.

Although there are some liquid chromatography coupled to mass spectrometry systems that are CE-IVD (i.e., European directive for *In Vitro* Diagnostics) approved for clinical biochemistry, toxicology, or therapeutic drug monitoring, and mass spectrometers can be used in operating rooms [139, 140], not all medical laboratories are and will be equipped with mass spectrometers (especially high-resolution instruments) and very few of them with nuclear magnetic resonance instruments. Thus, other analytical methods, such as enzyme assays, immunoassays, and biosensors, have to be envisaged for metabolite detection. For example, enzyme- and immunoassays are already widely and routinely used in hospitals for clinical biochemistry and therapeutic drug monitoring, as part of industrial automated in vitro diagnostic systems.

## Alternative tools to mass spectrometry and nuclear magnetic resonance instruments for making metabolomics valuable in clinical laboratories

### Enzyme assays for monitoring metabolites in biological fluids

As previously emphasized, enzyme assays are already widely used in clinical laboratories for monitoring metabolites, such as bile acids [141], formic acid [142], oxalic acid [143], or sialic acid [144]. Such assays are mainly based on the monitoring of enzymatic cofactors such as NADH or NADPH that are consumed by the enzymatic reaction together with the analyte. Although simple to implement when kits are commercially available, these approaches may suffer from low specificity and may lead to underestimated values, as recently reported for bile acids [145]. They are also prone to interferences, as observed with oxalic acid for which vitamin C interferes [143]. Furthermore, as enzymes may process all the members of a chemical class, some enzyme assays enable the determination of total concentrations rather than individual ones, as observed for bile acids and triglycerides, for example. Finally, such assays suffer from low multiplexing capabilities.

For all these reasons, if enzymatic methods are useful to analyze a metabolite or possibly a set of metabolites belonging to a given chemical class, it seems unlikely to use them for more complex metabolomics signatures as they require the ability to find and produce dedicated enzymes and to set up conditions allowing enzymatic activity measurement in different types of biological media.

### Laboratory immunoassays for the detection of metabolites in biological fluids

As main tools of immunoassays, antibodies enable the detection and quantification of specific biomarkers and are particularly suitable for molecules with a molecular weight above 3000 Da [146]. Performances of an immunoassay will rely first on the affinity and specificity of the antibodies used: polyclonal antibodies (i.e., collection of purified immunoglobulin molecules obtained from immunization of animals such as rabbits, sheep, donkeys, or goats), usually easier and faster to produce than monoclonal antibodies (i.e., single class of antibodies produced by a monoclonal immortalized B lymphocyte), generally display high affinities for their target but limited specificity. Monoclonal antibodies (mAb) are preferred tools for the development of accurate and specific immunological tests. Second, performances will depend on the detection system that can be achieved using various methods, including among others isotopic labeling (radioimmunoassays), enzyme reaction with UV-visible or fluorescence or chemiluminescence detection, or colloidal gold particles.

There are two main immunoassay formats: competitive and immunometric, depending on the size of the analyte. In immunometric assays, a first capture antibody, specific for the antigen, is bound to a solid surface. The antigen is then added, followed by addition of a detection antibody. The latter binds the antigen to a different epitope from the capture antibody. Thus, this assay format is adapted to analytes containing a least two epitopes (i.e., molecules having a molecular mass above 1000 Da, assuming that an epitope includes at least 5 amino acids). Conversely, in competitive immunoassays, the antibody is immobilized on a support and the detection is achieved through a labeled antigen. The addition of the sample containing free antigen induces an antibody binding equilibrium between the free antigens and the labeled ones. This assay format is preferably used when only one antibody is available, or when the analyte has only one epitope (i.e., small antigen < 1000 Da), which is the case for metabolites.

Before the development and popularization of LC-MS approaches in the 1990s, competitive immunoassays were widely used for the sensitive detection of drugs and their metabolites for pharmacokinetics and therapeutic drug monitoring, and for clinical chemistry, as it was for example the case for steroid hormones [147, 148]. Nowadays, competitive immunoassays are still used for a number of clinical biology analyses such as the detection of 25-hydroxyvitamin D (vitamin D metabolite) by radioimmunoassay [149], the detection of oxidative stress biomarkers based on the combination of microfluidics and fluorescent immunoassay [150], and the detection of hormones such as progesterone by coupling microchip electrophoresis and chemiluminescent immunoassay [151] or cortisol using a paper-based immunosensor with a colloidal-gold labeled immunoassay [152].

**Fig. 5:**
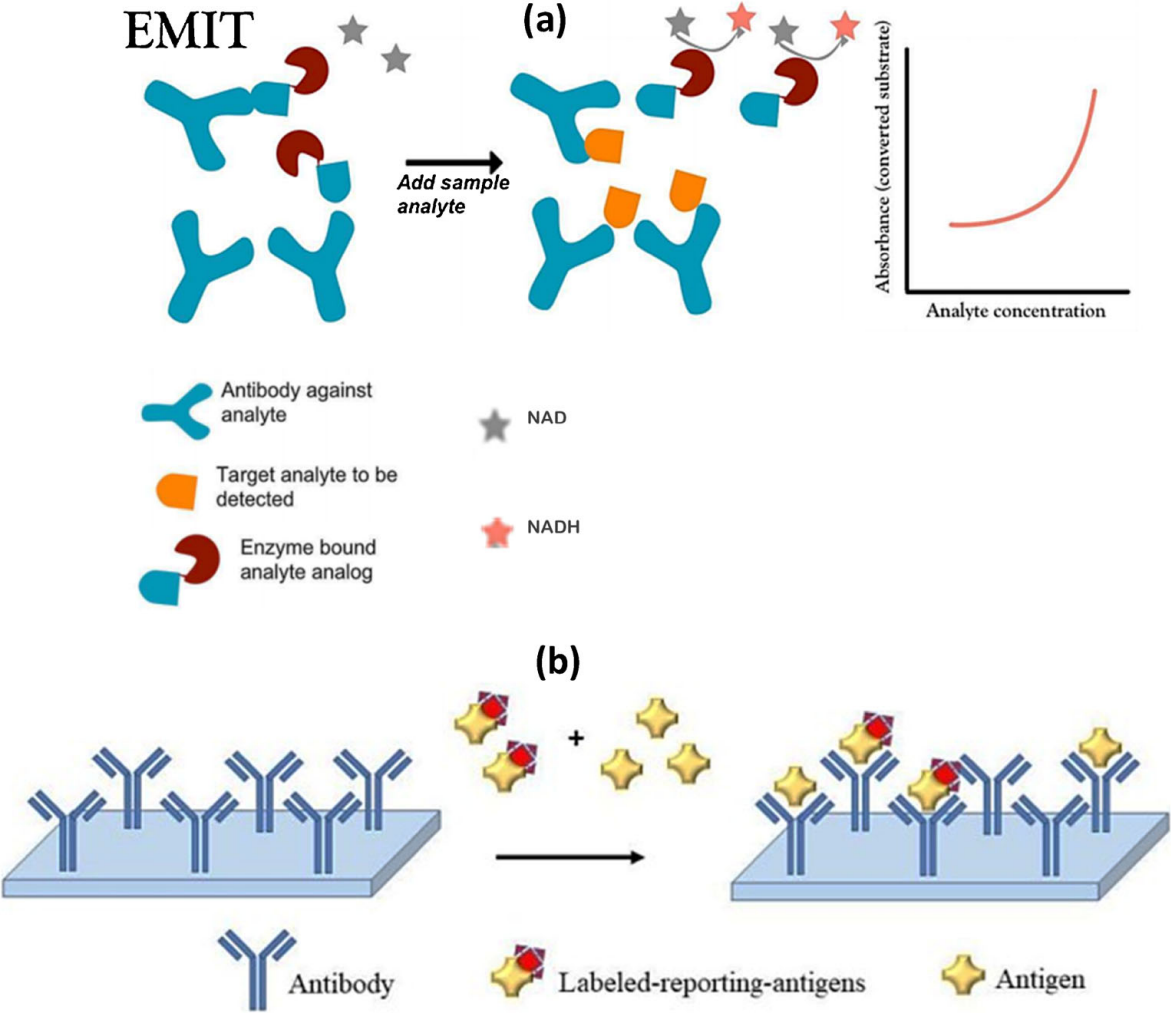
Competitive laboratory immunoassays for small molecules. **a** Principle of EMIT (Enzyme Multiplied Immunoassay Technique) [153]. EMIT is a competitive immunoassay in homogenous phase in which an analyte analog is bound to an enzyme using nicotinamide-adenine-dinucleotide (NAD) as a cofactor. The enzymatic reaction generates NADH which is detected by spectrophotometry at 340 nm. A competition between the analyte and the enzyme bound analog takes place toward the antibody. The amount of NADH produced is directly related to the amount of analyte present in the sample. **b** Competitive ELISA [154]: Antibodies are immobilized on the solid support. A competition takes place between an analyte analog coupled to an enzyme and the free analyte in the sample. The detection is achieved through enzymatic activity

Various types of competitive assays have thus been developed and implemented on automated in vitro diagnostic devices. The most popular are competitive immunoassays in homogeneous phase (i.e., reagents, samples, and measurements are achieved in a liquid phase), such as EMIT (Enzyme Multiplied Immunoassay Technique; see Fig. 5a), CEDIA (Clone Enzyme Donor Immunoassay), FPIA (Fluorescence Polarisation Immunoanalysis), and KIMS (Kinetic Interaction of Microparticle in Solution) [153].

Laboratory immunoassays can also take place in heterogeneous phases. In this case, the assays are performed in several steps with reagents added and optionally washed or separated at the site of the antigen/mAb complexes. Enzyme linked immunosorbent assays (ELISA) are probably the most used heterogeneous phase assay formats for the detection and quantification of biomarkers in biological media. Since it requires multiple steps (and washing steps in between), ELISA (Fig. 5b) needs to be performed by trained staff with laboratory equipment.

**Fig. 6:**
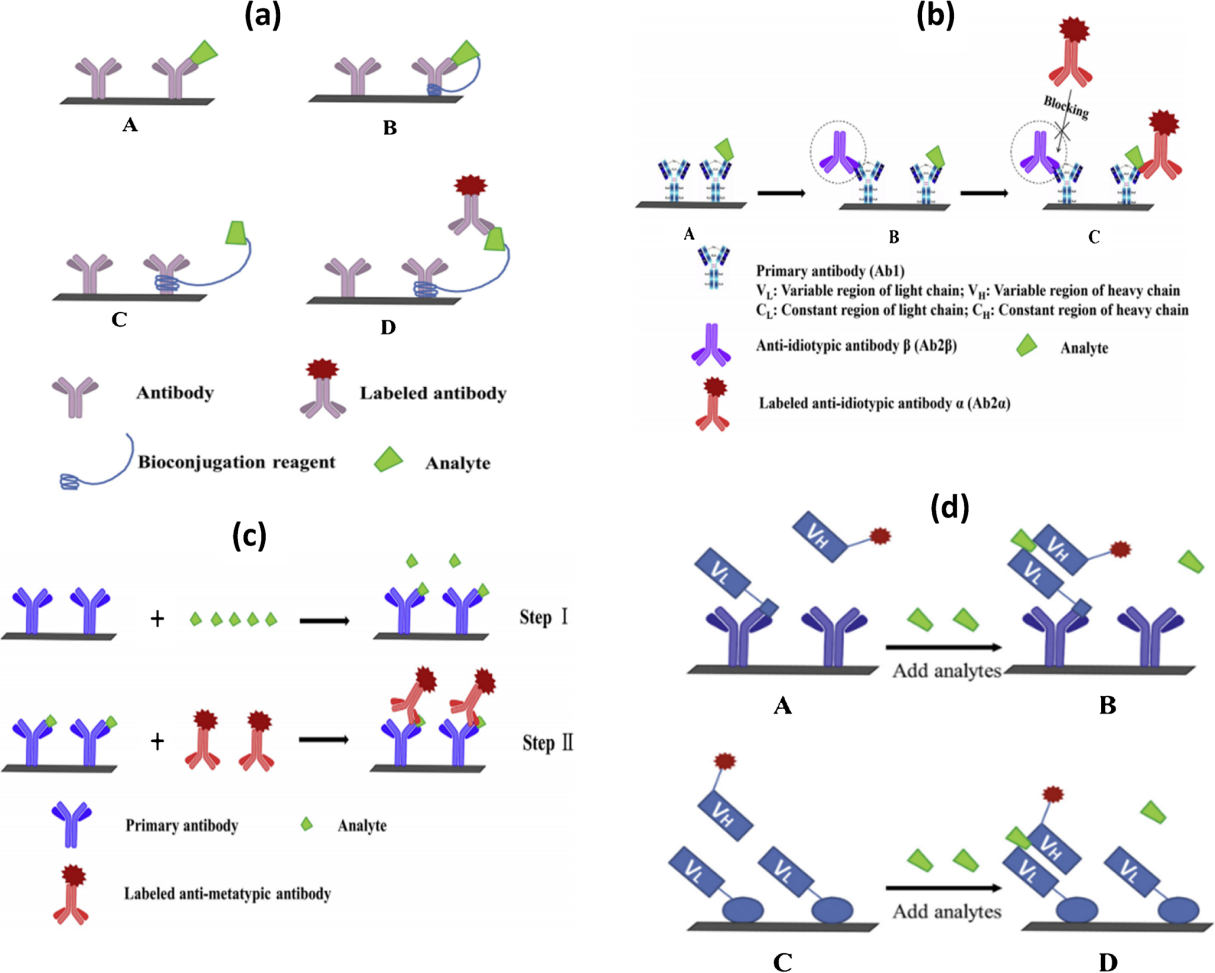
Non-competitive laboratory immunoassays for small molecules [155]. **a** SPIE-IA (solid-phase immobilized epitope-immunoassay): This format is based on the use of a single antibody that acts as both capture and detection antibody. It takes place in four steps: (i) Analytes are captured by immobilized antibodies. (ii) Analytes are covalently bound to the immobilized antibodies with the help of a reagent (e.g., glutaraldehyde, carbaonyldiimidazole). (iii) C-Analytes are then released from the immobilized antibodies by denaturation with a solvent. (iv) Detection antibodies coupled to an enzyme can then fix the analytes. **b** AIA-NIA (anti-idiotypic antibody-based non-competitive immunoassay): This format requires the use of three antibodies: an immobilized primary antibody (Ab1), an anti-idiotypic antibody (Ab2α), and a labeled anti-idiotypic antibody (Ab2β) and is performed in four steps : (i) The analyte binds to Ab1. (ii) Ab2β is added to block the remaining Ab1 free binding sites. (iii) Ab2α are then added to capture only the Ab1/analyte complexes (Ab2β/Ab1 complexes cannot be captured due to steric hindrance). The signal strength is proportional to the amount of Ab2α labeled and bound to the Ab1/antigen complex. **c** AICA-NIA (anti-immune complex antibody-based non-competitive immunoassay): This assay uses an immobilized (Ab1) and an anti-metatypic (Ab2) antibody, the latter stabilizing the antibody/analyte complex. It takes place in two stages: (i) The analyte binds to the Ab1. (ii) Ab2 is added and binds the analyte-antibody complexes. The intensity reflects the amount of Ab2 that has bound. **d** OS-NIA (open sandwich non-competitive immunoassay): This format is based on the association of free VH and VL chains from the variable domain of an antibody, which dissociate in the absence of the antigen (i.e., the analyte). It takes place in two stages: (i) The VL chains, conjugated to a carrier protein, are fixed by immobilized antibodies. (ii) The analyte and the labeled VH chains are added. The binding of the antigens to the VL chains allows the association of the VL and VH chains. The intensity of the signal is proportional to the quantity of labeled VH chains present

Finally, other types of assays aiming at overcoming the limitations of competitive formats have been specifically developed for the detection and quantification of small molecules. Some of them, such as SPIE-IA (solid-phase immobilized epitope-immunoassay), AIA-NIA (anti-idiotypic antibody-based non-competitive immunoassay), AICA-NIA (anti-immune complex antibody-based non-competitive immunoassay), and OS-NIA (open sandwich non-competitive immunoassay), are described in Fig. 6 [155].

Although some of these assay formats are attractive and inspiring for the design of biosensors for personalized medicine purposes, immunoassays for small molecules suffer from several limitations. Indeed, the lack of sensitivity and specificity of antibodies for small molecules are their main weaknesses. Although many antibodies have been developed against small molecules, they can suffer from cross-reactions. This is the case, for example, for antibodies against cortisol which have cross-reactivity with many endogenous steroids such as cortisone or many synthetic steroids such as prednisolone or prednisone [156]. The challenge to develop assays based on the recognition of metabolites by antibodies firstly lies in the possibility to derive these small chemical molecules, to make them haptens (i.e., to link them covalently to carrier proteins whose role is to provoke/favor the immune response in immunized animals, naturally not immunogenic). In some cases, complex coupling chemistry procedures are necessary to obtain an immunogen [157]. Furthermore, antibodies of interest have to be selected on their specificity to recognize exclusively the molecule, without recognition of the derived molecule, or of related molecules. The smaller the molecule, the more difficult this selection is to achieve. Competitive immunoassays also exhibit poorer sensitivity than immunometric ones because of the use of a limited amount of reagent, which does not facilitate the formation of antigen/antibody complexes.

Finally, there could be issues of loss of performance, potentially in terms of specificity, when moving from mass spectrometry–based methods to immunoassays. In this context, there are many studies aimed at comparing the performances of immunoassays and mass spectrometry–based approaches. Most of them deal with drugs and their metabolites, and, to a lesser extent, with endogenous metabolites such as steroids, thyroid hormones, and vitamin D derivatives. Some of these studies show that these two methods exhibit the same kind of performances [158–160], whereas others point out variable quantitative performances between immunoassays [161], and specificity issue with over- or underestimated concentrations measured in immunoassays [162–165].

Anyway, despite these limitations, it is important to continue working on new small-molecule immunoassay formats given their sensitivity, their relative simplicity of implementation, and the lack of efficient alternative methods, when methods based on mass spectrometry and proton nuclear magnetic resonance spectroscopy cannot be used.

### Biosensors for small molecules: toward point of care tests for metabolomics?

**Fig. 7:**
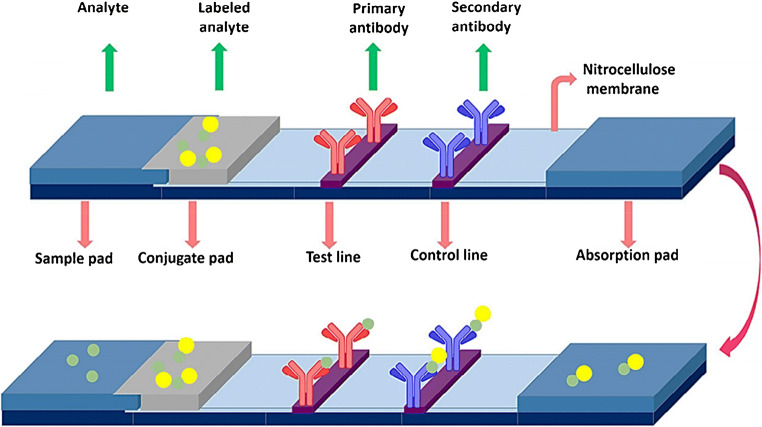
Principle of competitive lateral flow immunoassays: The device is composed of four parts: (i) a sample pad, on which the sample is deposited; (ii) a conjugate dried buffer, containing the labeled analyte analog; (iii) a nitrocellulose membrane, on which are found test line(s) composed of antibodies recognizing the analyte(s), and control line(s) formed by antibodies which recognize the labeled analyte analog; and (iv) an absorbent paper, which serves to pump the liquid sample and reserves any excess sample. In the absence of the target analyte (negative sample), labeled analog analytes move through the strip and bind on both test and control lines. In a positive sample, a competition takes place on the test line between the analyte and its labeled analog. As before, the excess of labeled analyte analog is captured by antibodies in the control line. Thus, a signal is only observed on the control line. Adapted from reference [166]

Point-of-care (PoC) tests are medical laboratory diagnostic tests intended to be carried out in the direct proximity of the patient, at the physician’s office, in pharmacies, in medical centers, in the emergency rooms of hospitals, or even in professional laboratories. Whether in hospitals or not, PoC tests are expected to give results within a short period of time (30 min). Those tests thus require the smallest possible logistical footprint, in terms of transport and space and storage conditions, as well as handling time, and are designed to be carried out by staff not necessarily trained in laboratory medicine (nurse, medical assistant), or even by the patient himself or his relatives. Lateral flow immunoassay (LFIA) is probably the ideal on-field point-of-care test since it does not require complex sample preparation or further steps after the deposition of the sample and can be performed easily by untrained staff. Although mainly used for protein targets, competitive LFIA, whose principle is described on Fig. 7, have been developed for small molecules. However, they are more complex to implement and less sensitive than non-competitive LFIA for different reasons. Firstly, obtaining a specific and affine antibody is difficult as the molecule is small [146]. Secondly, the decreased signal observed in competitive methods is more difficult to interpret than the appearance of a signal [146]. Moreover, these tests are neither suitable for multiplexed detection nor for field quantification, and are subject to matrix effects. Indeed, in the case of small endogenous molecules, the challenge is important: some of them may have relatively small variations (less than a factor of 5) between a physiological and a pathological state, they generally require a preliminary sample extraction step, and only the integration of data from several biomarkers will allow the interpretation of a biological signature. The test must be accurate, robust (with little matrix effect), quantitative, highly multiplexable, and easily interpretable, and must integrate automated sample preparation before analysis. Therefore, the PoC tests for fine, multiplexed, integrated quantification of multiple metabolites of different natures must address unmet needs and will require a large research effort which will be driven by the need of clinicians [167].

Research on rapid tests has developed considerably, especially regarding nanoparticles-based devices [168] and microfluidics [169], paving the way to the emergence of new diagnostic devices [170]. Wearable chemical sensors enabling real-time on-body analytical chemistry are in increasing development. However, they only allow monitoring electrolytes and few metabolites such as glucose or lactic acid at the time of writing this review [171].

Biosensors are devices that combine the physical properties of a transducing platform (electrical, optical, etc.) with those of a biological or synthetic component in order to detect, i.e., sense, the presence and, possibly, quantify the concentration of a target compound, i.e., the analyte. This combination, if properly addressed, benefits from both the natural selectivity of a wide range of bioreceptors (enzymes, antibodies but also nucleic acids and even viruses, bacteria, and whole cells) or synthetic molecules (i.e., molecularly imprinted polymers (MIPs), aptamers, etc.) and the sensitivity of the devices converting, i.e., transducing, the binding event between the bioreceptors and the analyte in an analytical signal. Depending on the nature of this signal, several transduction methods could be employed, such as optical, electrochemical, thermal, or piezoelectric biosensors.

Biosensors are one of the most important representatives of PoC devices that are having a remarkable impact on patients’ life and, thus, are also being pursued with the highest interest by the research community. Indeed, PoC biosensors’ characteristics should reach the criteria “REASSURED,” i.e., *r*eal-time connectivity, *e*ase of specimen collection and environmental friendliness, *a*ffordable, *s*ensitive, *s*pecific, *u*ser friendly, *r*apid, *e*quipment free, *d*elivered [172], as they should provide real-time connectivity and easy specimen collection.

Nanotechnology can push even further the characteristics of PoC biosensors by introducing nanomaterials, guaranteeing numerous advantages, such as ultra-high sensitivity, further miniaturization if necessary, nanoreceptors with high stability and specificity, and innovative detection mechanisms, improving the performances while decreasing the associated costs.

Most of the challenges of small-molecule detection are directly related to the use of biorecognition elements. As previously emphasized, due to the low molecular weight and simple structure of small molecules, they show a lack of immunogenicity when it comes to antibody production. For this reason, there have been strong efforts to develop other types of bioreceptors in order to have better performance with this type of molecules. Among them are aptamers and MIPs, which can be successfully applied to PoC nanobiosensors.

Aptamers are nucleic acid (DNA or RNA) short sequences that bind to a specific target and are determined through sequential evolution of ligands by exponential enrichment (SELEX) [173, 174]. A pool of random nucleic acid sequences is repeatedly incubated with the target analyte, and the unbounded sequences are discarded. After various rounds with different stringencies, the sequences with higher affinity for the target are selected. The SELEX methodology has been successful in developing aptamers for small molecules. This is in part thanks to the flexibility of the method, which can be adapted to the nature and needs of the target molecule (capture-SELEX, capillary-SELEX, or nitrocellulose-SELEX, for instance). [175, 176]. Conventional and capture-SELEX have been reported as suitable options for small molecules [177]. Theoretically, these methods allow the production of big amounts of aptamers at low cost and take less time compared to antibody production, as well as avoiding the use of animal testing and batch-to-batch variations. Of course, this allows the production of aptamers for toxic molecules that would kill the animal in the immunization process. Moreover, stability and affinity toward the target can be modulated by modifying the sequence of the aptamer, along with adding other elements to the sequence depending on its intended use (fluorophores, biotin, thiol groups, etc.), which is especially interesting for assays combining aptamers with nanomaterials. As SELEX is performed by incorporating negative counter-selection rounds, a high specificity and selectivity are ensured. In contrast, antibodies often show cross-reactivity between similar molecules [178].

**Fig. 8:**
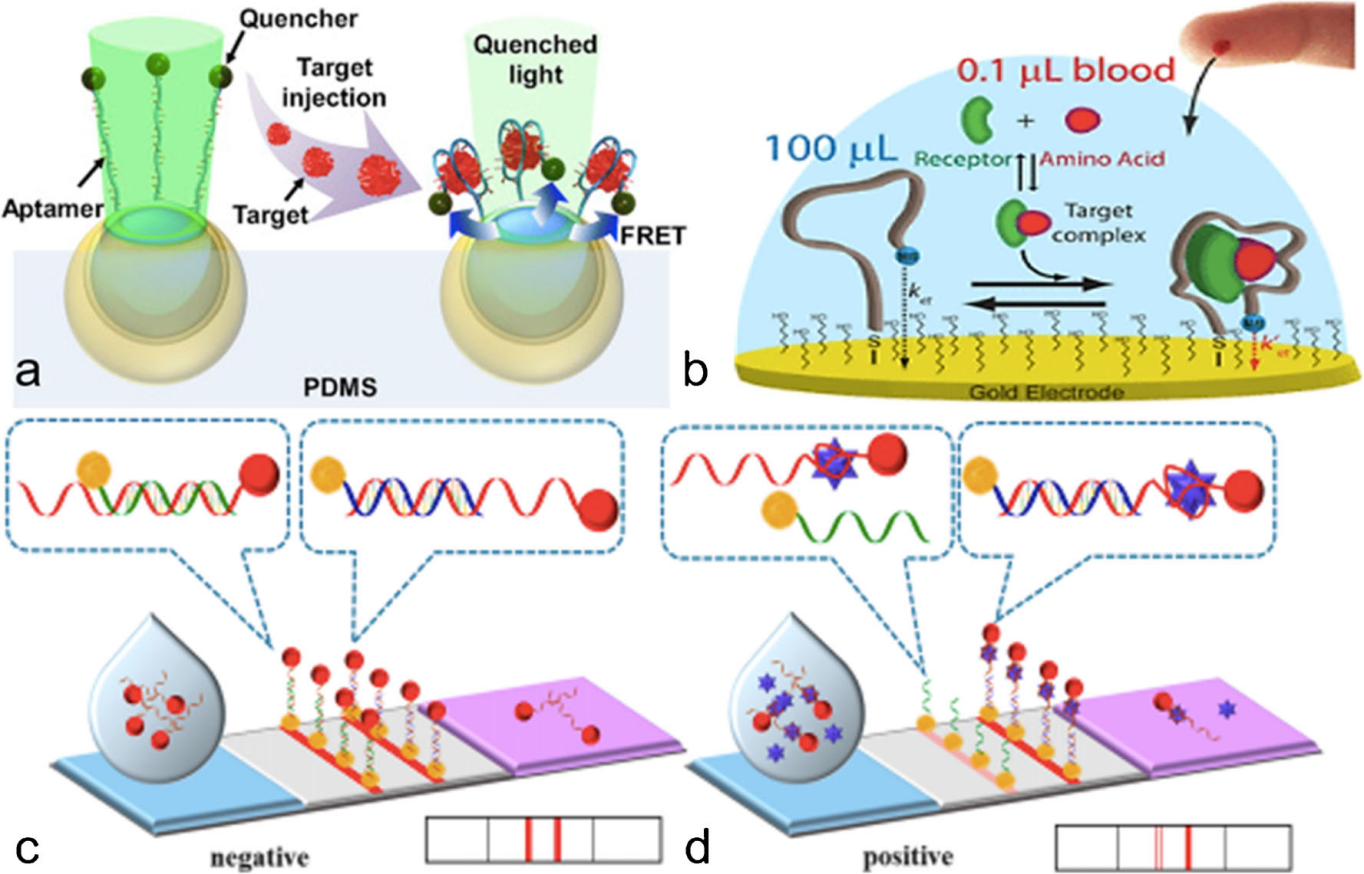
Overview of the possible adaptation of aptamers in different detection platforms combined with nanomaterials. (a) Gold nanocap-supported up-conversion nanoparticles for fabrication of a solid-phase aptasensor for ochratoxin A detection. Extracted from reference [209]. (b) Calibration-free measurement of phenylalanine levels in the blood using an electrochemical aptamer-based sensor suitable for point-of-care applications extracted from reference [210]. (c and d) Aptamer-based lateral flow test strip for rapid detection of zearalenone in corn samples. Adapted from reference [211]

Aptamers are approximately one tenth of the size of an antibody, with enough recognition surface area to target small molecules, unlike antibodies, which rely on epitopes that are often scarce in small molecules [179]. Compared with antibodies, the small size of aptamers also allows a high immobilization rate at the surface of a biosensor, increasing its sensitivity. Besides, they can be regenerated for long-term monitoring and are not susceptible to irreversible denaturation, in contrast to antibodies. Moreover, aptamers have a long shelf-life and stability under strong conditions such as temperature, chemicals or pH, which makes its transportation, storage, and performance much easier in remote areas [178]. As depicted in Table 3, different platforms and detection strategies have been successfully and sensitively detecting small molecules in biological samples by using aptamers in the last decade. For instance, microfluidic devices, lateral flow and electrochemical systems are some of the potential platforms where aptamers can be implemented (see Fig. 8).

**Table 3:** Characteristics and performance of the main biosensors currently available for small-molecule detection in biological fluids

**Analyte**	**Transducer**	**Bioreceptor**	**LOD**	**Response time**	**Biological fluid**	**Ref.**
Cocaine and synthetic cathinones	Colorimetric	Aptamer	10 μM	5 min	Saliva and urine	Luo et al., 2019 [198]
Cocaine	Microfluidic, electrochemistry	Aptamer	10 μM	1–2 min	Blood serum	Swensen et al., 2009 [199]
Adenosine trihosphate (ATP)	Lateral-flow assay	Self-assembly of split aptamers fragments	2 μM	10 min	Blood serum	Chen et al., 2012 [200]
Adenosine	Electrochemistry uPAD	Aptamers	5.7 μM	10 min	Urine	Fu et al., 2017 [201]
Cocaine, ATP	Fluorescence	Exonuclease-mediated aptamer digestion	500 nM	25 min	Urine	Canoura et al., 2018 [202]
Tetrahydrocannabinol	Magnetoresistive sensor	Antibodies competitive detection	10 ng/ml	< 15 min	Saliva	Lee et al., 2016 [203]
Ochratoxin A, aflatoxin B1, ATP, potassium ions	Localized surface plasmon resonance	Aptamers on gold nanorods	0.56, 0.63, 0.87, 1.05 pM	30–60 min	Serum	Park et al., 2017 [204]
l-Tyrosinamide	Fluorescence polarization assay	Aptamer	200 nM	< 10 min	Urine	Ruta et al., 2009 [205]
Phenytoin	CMOS BioMEMS		4.06 μg/ml	25 min	Artificial samples	Yen et al., 2020 [206]
Dopamine, cortisol, serotonin	Thermal variation	MIPs and thermal transducers	8 μM	/	Serum and urine	Diliën et al., 2017 [186]
Carnitine	Potentiometric	MIPs, Radical polymerization	80 μM	/	Urine	Moret et al., 2014 [187]
Dopamine	Ratiometric electrochemical	MIPs and nanoporous Au	0.1 μM	2 min	Artificial CSF	Yang et al., 2019 [207]
Glucose	Electrochemial	MIPs and AuNPs	1.25 nM	30 min	Serum	Sehit et al., 2020 [188]

To summarize, aptamers have shown potentially great advantages over the use of antibodies for diagnostic applications since their discovery in 1990. This is especially of interest for small molecules for which antibodies are not well suited. However, their implementation into PoC nanobiosensors is rather scarce [169], and despite promising results, there is still no commercialized diagnostic tests based on aptamers. Studies are needed to evaluate their performance in biological media for diagnostic tests involving metabolites.

For the last decades, it has been a challenging task to generate polymeric matrices with homogeneously distributed and highly specific cavities, thoroughly designed to match the dimensions and chemical functionality of a target molecule. This type of bioreceptors is known as molecularly imprinted polymers (MIPs), which are considered synthetic biorecognition elements resulting from the polymerization of a precise combination of one or several functional monomers and the desired target, i.e., “template molecule,” along with other components present in the polymerization solution, such as a cross-linker molecule. The utmost important challenge that needs to be addressed in the first stages of the development of a MIP would be to study the binding interactions among the monomers and between the monomer and the template, which must be guaranteed in order to form a spontaneous and stable template-monomer complexation. From this, several other parameters must be considered for the formation of a single-molecule MIP or a “class-selective” one, which is particularly designed to detect a family of related molecules. Some of them are the template-to-monomer (T:M) ratio, the crosslinking degree, porogenic solvent selection, and some physical parameters, such as temperature, pH, and agitation of the polymer solution.

The use of MIPs as another metabolomics tool has been considered an interesting approach due to their fast production, robustness, chemical inertness, cost-effectiveness, and long-term stability. If precisely tuned, they can withstand extreme values of pH and temperature [180, 181]. Unlike current and typical immunoaffinity-based approaches, MIPs have demonstrated a higher sample load capacity for small molecules (MW below 3 kDa), resulting in higher recoveries for further analytical applications, as well as displaying a slightly higher selectivity and specificity toward smaller targets [182].

**Fig. 9:**
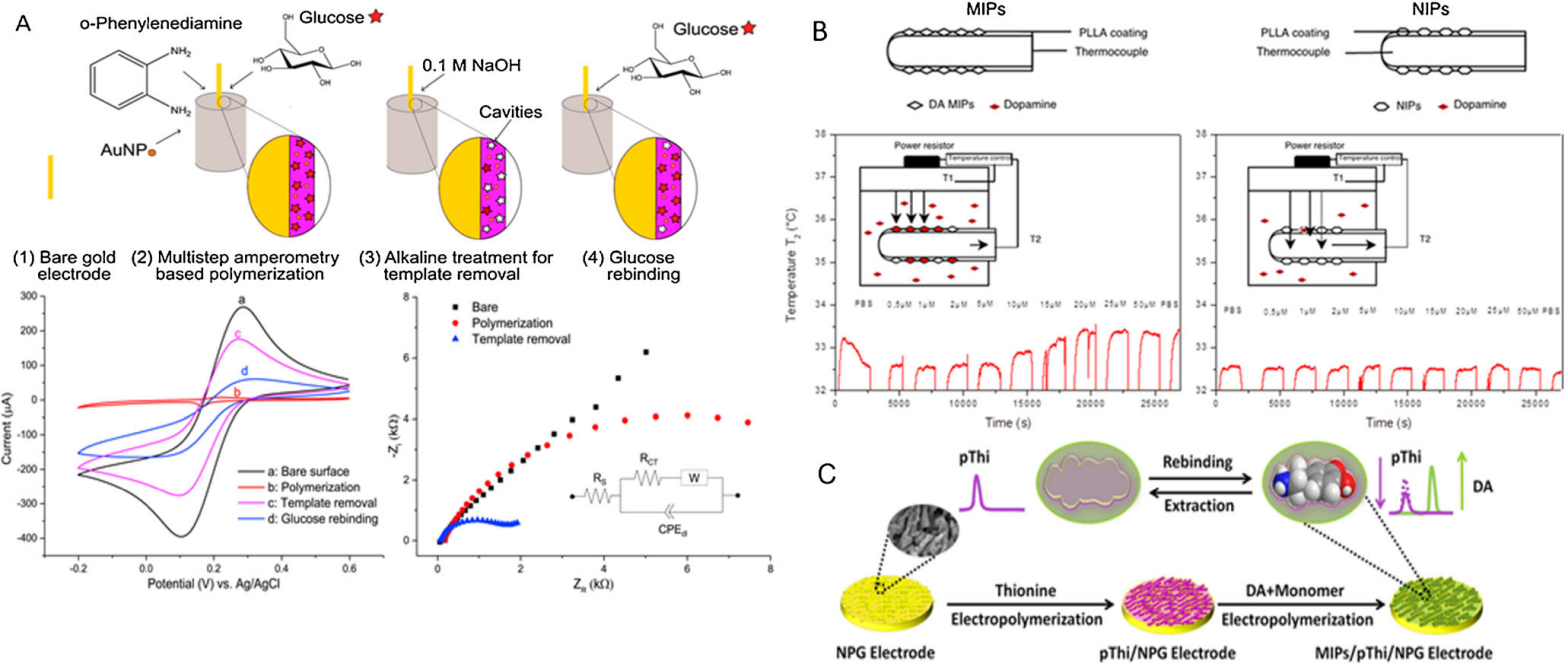
MIP-based biosensing platforms. **a** Gold nanoparticle (AuNP) decorated MIPs using o-PD and glucose as monomer and template molecules, respectively. CV and DPV measurement of each step. Extracted from Sehit et al. [188]. **b** Schematic representation of the heat flow through the MIP and NIP-coated thermocouples. Extracted from Diliën et al. [186]. **c** Schematic diagram of the synthesis process of MIPs/pThi/NPG electrodes, along with their respective DPV measurements and the calibration curves for dopamine detection. Adapted from Yang et al. [207]

Based on the final applicability of the desired sensor, several approaches have been developed. For detecting tamoxifen, for example, an estrogen receptor used to prevent breast cancer, a successfully electropolymerized MIP composed of o-phenylenedediamine and resorcinol was developed [183]. This methodology demonstrated a better diffusion rate, permeability, and binding affinity to the target. Acetaminophen has also been investigated for medical and clinical purposes, by using signal enhancers, such as gold nanoparticles [184], or developing a thin MIP layer that enhances the selectivity [185]. Other metabolites have also been studied, such as dopamine [186], carnitine [187], and glucose [188], among others, as it can be seen in Table 3 and Fig. 9.

Anyway, to our knowledge and as for aptamers, there are still no commercialized diagnostic tests based on MIPs. These two types of synthetic bioreceptors (aptamers and MIPs) need to address the issue of properly working in real biological fluids, as well as for enzyme- and immunoaffinity-based systems, as it was aforementioned [189]. Most metabolites are often found in ultra-low concentrations in biological samples. In these cases, the integration of nanomaterials clearly upgrades the sensitivity, either by signal amplification or better immobilization of the biorecognition elements [190]. Another inconvenience is the complexity of biological fluids, which might contain contaminants causing cross-reactivity, or even nucleases, that are detrimental for these bioreceptors [191]. While electrochemical platforms are well known for their advantages in complex matrices like blood (i.e., glucometer, the most-known example), optical platforms still face the problem of interferences mainly due to the viscosity and optical properties of the samples (e.g., the deep red of blood causes background interference) [192] [193].

## Conclusion

Metabolomics workflows, including sample preparation, MS and/or NMR analyses, data pre-processing, statistical analyses, and data visualization, have been developed since the 2000s and have now reached a certain level of maturity. Data production methods have gained in sensitivity and versatility, suggesting the possibility of achieving metabolite detection, identification, and quantification at the same time. Many guidelines covering pre-analytical stages, data acquisition, and study design have been published. Quality management processes have been proposed, become consensual, and are more and more used. Data warehouses dedicated to metabolomics have been developed, improving data sharing. Despite this, no diagnostic test based on metabolomics has yet been marketed. The main issues are linked to lacks of standardized data production tools and interoperability, to inappropriate design of clinical trials for the discovery and validation of metabolomics signatures, and to the difficulty of integrating multiscale biological information to generate knowledge and predictive models.

However, more than 600 articles dealing with metabolomics for the investigation of medical cohorts have been published over the 2015–2020 period, some of them leading to proposed metabolomics signatures of disease diagnosis and severity, and response or non-response to treatments. In this context, it is now time to consider how to be prepared to efficiently transfer future metabolomics signatures to clinical settings. First of all, metabolomics signatures obtained from untargeted metabolomics cannot be directly used in the routine care practice. There is a need for simplification and for moving to quantitative results. Indeed, many actors and structures are involved in healthcare systems, such as clinical units in hospitals, medical laboratories in hospital settings or outside the hospitals, physician’s offices, pharmacists, and at least the patient at home.

A first step regarding the transfer of metabolomics signatures to the field could rely on medical laboratories in hospital settings. Clinical biologists will certainly have a key role in data production and interpretation, and for transmitting the results, clinicians by integrating the biological message into the global clinical context. In this case, one can think about centralized data production platforms equipped with (high-resolution) MS or NMR instruments. However, not all medical laboratories are and will be equipped with these instruments. Thus, there is a need to consider alternative methods, such enzyme assays, immunoassays, and biosensors. In particular, even if enzyme assays have been and are still widely used for monitoring small molecules in biological fluids, they are restricted to few key metabolic intermediates present at high concentrations, and they have low multiplexing capabilities. Immunoassays are also very popular in the field of clinical and environmental chemistry, regarding the detection of drugs and more generally xenobiotics. However, they still suffer from limitations linked to the difficulty of generating antibodies having satisfactory sensitivity and specificity, which limits the design of biosensors for small molecules. Aptamers and molecularly imprinted polymers are attractive and promising alternatives to antibodies, especially in the field of small molecules, but further research efforts are needed to evaluate their relevance in complex biological media.

To conclude, it is important to intensify research in analytical chemistry, not only in the generation of metabolomics data for producing interoperable and reusable data, but also in the field of point-of-care tests, in order to be ready when molecular signatures can be used in routine care practice. Furthermore, the small metabolite concentration variations that are often observed between groups in many metabolomics and lipidomics studies represent an important limitation for clinical translation, which will call for new ways of thinking in the fields of analytical chemistry and data sciences to overcome this issue.
